# Exploring the liver microenvironment following successful therapy for HCV: gene expression profiling and residual T cell infiltration

**DOI:** 10.3389/fcimb.2025.1662184

**Published:** 2025-11-10

**Authors:** Daniel E. Millian, Esteban Arroyave, Timothy G. Wanninger, Santhoshi Krishnan, Daniel Bao, Jared R. Zhang, Arvind Rao, Heidi Spratt, Monique R. Ferguson, Vincent Chen, Kellen Henning, Heather L. Stevenson, Omar A. Saldarriaga

**Affiliations:** 1 Department of Pathology, University of Texas Medical Branch, Galveston, TX, United States; 2 Department of Microbiology and Immunology, University of Texas Medical Branch, Galveston, TX, United States; 3 Department of Computational Medicine and Bioinformatics, University of Michigan, Ann Arbor, MI, United States; 4 Department of Electrical and Computer Engineering, Rice University, Houston, TX, United States; 5 John Sealy School of Medicine, University of Texas Medical Branch, Galveston, TX, United States; 6 Department of Radiation Oncology, University of Michigan, Ann Arbor, MI, United States; 7 Department of Biostatistics, University of Michigan, Ann Arbor, MI, United States; 8 Department of Biomedical Engineering, Rice University, Ann Arbor, MI, United States; 9 Department of Biostatistics and Data Science, University of Texas Medical Branch, Galveston, TX, United States; 10 Department of Internal Medicine, University of Texas Medical Branch, Galveston, TX, United States; 11 Department of Internal Medicine - Gastroenterology, University of Michigan, Ann Arbor, MI, United States

**Keywords:** hepatitis C virus, direct-acting antiviral, sustained virologic response, liver, hepatic microenvironment

## Abstract

**Background & aims:**

Direct-acting antivirals (DAAs) revolutionized hepatitis C (HCV) treatment. Yet, some patients present with persistent inflammation and still face adverse outcomes, including cirrhosis and hepatocellular carcinoma, despite achieving sustained virologic response (SVR).

**Approach & results:**

This study examined liver biopsies from 22 patients before and after DAA treatment to assess gene and protein expression changes linked to disease progression. Pre-treatment, patients exhibited two distinct profiles: one characterized by high pro-inflammatory and antiviral gene expression (pre-hot), and another with weaker expression (pre-cold). Patients with pre-hot profiles were initially associated with poor outcomes (OR = 14.0, 95% CI: 1.31–178.5; p = 0.049), but this lost significance after adjusting for baseline disease severity (adjusted OR = 8.04, 95% CI: 0.18–2123.07; p = 0.328). Baseline modified hepatitis activity index (MHAI) scores (OR = 1.90, 95% CI: 0.72–6.34) and fibrosis stage (OR = 1.65, 95% CI: 0.44–9.97) trended toward predicting poor outcomes but were not significant. Post-treatment, liver enzymes decreased, inflammatory scores improved, and type I interferon pathways were restored, yet 14 of 17 patients (82.3%, 95% CI: 64.2–100%) retained persistent lymphocytic infiltrates. In parallel, spectral imaging further revealed post-treatment shifts in macrophage populations, with a significant decrease in inflammatory subsets (CD14+, CD14+/Mac387+, p<0.05) and an increase in anti-inflammatory subsets (CD16+, CD16+/CD163+, CD16+/CD68+, p<0.05). Analysis of T cell–related marker expression before and after treatment shows that mixed lymphocytic infiltration (CD3+, CD4+, CD8+, CD45RO+) persists within the liver despite viral clearance, with high inter-patient variability likely limiting statistical significance.

**Conclusions:**

Even after achieving SVR and normalization of gene expression, the liver retained a heterogeneous T cell infiltrate, suggesting that persistent immune activity could continue to influence the risk of adverse outcomes.

## Introduction

Although hepatitis C virus (HCV) infection continues to be a global health challenge, with approximately 50 million infected globally and one million new cases each year, the landscape has shifted dramatically in recent years due to the availability of direct-acting antiviral (DAA) therapy (Burki, 2024). By selectively targeting enzymes essential for viral replication, DAAs have achieved cure rates higher than 90% (Oru et al., 2021). This has substantially reduced the number of patients progressing to chronic HCV, the occurrence and recurrence of hepatocellular carcinoma (HCC), and all-cause mortality, which represent significant milestones in the clinical management of patients infected with HCV (Mendizabal et al., 2020). However, despite many patients achieving sustained virologic response (SVR) after DAA treatment, many individuals in high-risk populations remain susceptible to reinfection due to the absence of an effective vaccine and the surge in HCV incidence driven by the opioid epidemic. Specifically, injection drug users account for approximately 70% of new HCV infections (Ko et al., 2019).

The liver is a unique immune organ enriched with a diverse array of myeloid and lymphoid cells, and it relies on tolerogenic mechanisms to maintain homeostasis. HCV infection disrupts this balance by triggering the activation of both innate (e.g., type I interferons and IFN-stimulated genes) and adaptive immune responses (e.g., helper and cytotoxic T cells), which play a crucial role in either mediating viral clearance or sustaining chronicity (Noureddin et al., 2015). Inflammatory macrophage activation and cytotoxic T cell responses are typically protective, as they help to control viral replication and eliminate infected cells. However, the impairment of inflammatory macrophages and T cells, the induction of alternatively activated macrophages involved in tissue repair processes, and the promotion of exhausted T cells (characterized by a loss of effector functions and sustained expression of inhibitory receptors) contribute to HCV persistence in the liver (Saha et al., 2016; Ahmed et al., 2019; Barili et al., 2020). Human immunodeficiency virus (HIV) coinfection exacerbates HCV-induced inflammation and impairs both the innate and adaptive immune responses against HCV (Abutaleb and Sherman, 2018). DAAs have shown the potential to reverse these mechanisms and boost anti-HCV immunity.

In some cases, patients treated with DAAs exhibit persistent inflammation in the portal tracts of the liver without changes in the fibrosis stage or features of regression (Hsieh et al., 2021). Post-treatment lymphocytic inflammation, distinguished from other types of active hepatitis by its confinement to the portal tracts without interface and lobular activity, can still pose a diagnostic challenge for clinicians and pathologists managing HCV patients (Saldarriaga et al., 2021). Persistent lymphocytic portal inflammation is frequently observed in allograft liver biopsies after treatment, which can be puzzling and create uncertainty about whether the virus has truly been eliminated, despite clinical evidence of SVR (Whitcomb et al., 2017). Moreover, it is unclear whether patients who achieve SVR but exhibit histological evidence of persistent lymphocytic inflammation are predisposed to adverse outcomes, such as cirrhosis or cancer. Advanced imaging and single-cell platforms have been used to enhance understanding of changes in both the cellular composition and spatial distribution within the hepatic microenvironment (MacParland et al., 2018). However, studies of the liver after DAA treatment in the absence of HCV are limited, in part because the current standard of care no longer includes pre- or post-treatment liver biopsies, thereby reducing the availability of tissue for analysis (Bhattacharya et al., 2023).

This study aimed to analyze gene and protein expression changes in the hepatic microenvironment of patients before and after DAA treatment for HCV. It is unique in its access to liver biopsies from patients’ post-treatment, a resource that is rarely available under current clinical practice. This allowed us to correlate individual gene and protein signatures detected in the hepatic microenvironment with clinical outcomes and disease progression.

## Materials and methods

### Study design and patient inclusion criteria

We evaluated liver biopsies collected from adult patients before (i.e., pre-treatment) and after (i.e., post-treatment) DAA therapy for HCV, at the University of Texas Medical Branch (UTMB), between January 2008 and June 2021. This study was approved by the UTMB Institutional Review Board (IRB#13-0511), and all patients provided informed consent for research use of their tissue samples. The first biopsy was collected for diagnostic purposes prior to DAA treatment, while post-treatment biopsies were obtained only from patients who consented, as biopsy is not standard of care after SVR. The cohort included 22 patients: Seventeen patients with paired pre- and post-treatment biopsies, five with only pre-treatment biopsies, and one with only a post-treatment biopsy. Samples (pre and post) were matched by patient identifiers to allow longitudinal comparison. Demographic data, HCV viral load, and laboratory tests were recorded for all patients. No participants under 18 met the inclusion criteria.

### Control group criteria

Controls were individuals without liver disease, selected to provide a healthy baseline for gene expression profiling. They had no history of HCV or other hepatotropic infections, HIV, excessive alcohol/substance use, metastatic liver cancer, or immune-mediated liver conditions. Their biopsies showed no histopathologic abnormalities and liver enzymes were within normal ranges; however, control patients were not matched (**Supplementary Table S1**).

### Biopsy collection and histological assessment

Liver biopsies were obtained as part of standard care by licensed radiologists and processed in a College of American Pathologists (CAP)-accredited laboratory as described previously (Saldarriaga et al., 2021) and evaluated by a board-certified liver pathologist (H.S.L) blinded to the patient’s serology and clinical history. Histologic features were scored using the modified hepatitis activity index (MHAI) for inflammatory activity, Ishak staging for fibrosis, and steatosis grading (Ishak et al., 1995). Digital images were acquired using an Aperio ImageScope scanner. Adverse clinical outcomes, including HCC, were diagnosed based on standard clinical practice, typically using imaging modalities, in line with established guidelines. MELD scores were used to assess overall liver disease severity, not to confirm HCC diagnosis. Tissue blocks were stored at room temperature and sectioned before staining; unstained slides were stored at -80 °C when necessary.

### DAA treatment regimen and response to therapy

Patients received DAA regimens according to international guidelines for up to 12 weeks. Only those who achieved SVR were included. No patients had serologic evidence of coinfection with other hepatotropic viruses (e.g., hepatitis A, B, or E) or immune-mediated liver disease at diagnosis or during the study.

### Laboratory tests

Pre- and post-treatment laboratory tests (alanine aminotransferase [ALT], aspartate aminotransferase [AST], alkaline phosphatase [ALP], albumin, bilirubin, platelets, prothrombin time [PT], and international normalized ratio [INR]) were performed for all 22 patients. Viral load, genotype, and liver enzyme levels were assessed using CAP- and Clinical Laboratory Improvement Amendments (CLIA)-accredited molecular tests at baseline, three to four months after DAA treatment, and during follow-up.

### RNA isolation and nCounter gene expression analysis

RNA was extracted from formalin-fixed, paraffin-embedded (FFPE) and fresh liver biopsies, ensuring strict quality criteria (DV300 > 50%, 260/280 ≥ 1.7). Gene expression profiling was performed by hybridizing 50–150 ng of RNA with the PanCancer Immune Panel (730 immunological, inflammatory, and anti-viral targets and 40 housekeeping genes) on the nCounter platform (NanoString, Bruker Spatial Biology, Seattle, WA, USA). Quality control, normalization, differential expression, and immune-related scoring were conducted using nSolver 4.0™ analysis software. Normalization was performed using the most stable housekeeping genes, identified by the geNorm algorithm based on pairwise variation analysis (26 of the 40 available housekeeping genes were retained)(**Supplementary Table S2**).

### Differential gene expression and bioinformatics analysis

Hierarchical clustering, principal component analysis (PCA), volcano plots, and Venn diagrams were used to compare gene expressions among HCV pre- and post-DAA groups and controls. Differential expression analysis was performed using nSolver (NanoString/Bruker), which models counts data using a negative binomial model (glm.nb function) and applies Wald tests for group comparisons. Fold changes were calculated as log2 ratios between groups, and p-values were adjusted for multiple comparisons using the Benjamini–Yekutieli (B-Y) method. Volcano plots were generated by plotting log2 fold change on the x-axis against -log10 adjusted p-value on the y-axis. Significance thresholds were defined as B-Y FDR < 0.05 with log2 fold change ≥ ± 0.6. PCA was performed on log2-transformed, mean-centered, and unit-variance scaled gene expression data. The percentage of variance explained by each principal component was calculated and is indicated on the PCA plots. Volcano plots and PCA were visualized using GraphPad Prism v.10 (GraphPad Software, San Diego, CA, USA). Functional enrichment analysis was performed using the Database for Annotation, Visualization, and Integrated Discovery (DAVID) tool with Reactome database integration. Immune cell composition was estimated using CIBERSORTx, which infers relative cell-type proportions by comparing expression data against the LM22 reference matrix (547 RNA-seq–derived genes). Analyses were run with 500 permutations and B-mode batch correction to enhance robustness. Normalized NanoString PanCancer Immune Panel data (730 genes) were deconvolved against LM22 using overlapping genes between the two platforms. In total, 181 genes overlapped, including canonical markers for major T-cell subsets and macrophage/monocyte lineages (Chen et al., 2018). This overlap ensured adequate immune-specific coverage, enabling reliable estimation of the targeted populations in this study.

### Multispectral imaging analysis

Two multiplex antibody panels were used, targeting macrophages (CD68, CD163, Mac387, CD14, CD16) and T cells (CD3, CD4, CD8, CD45RO, FoxP3) with DAPI counterstaining. Staining was conducted manually, or with the Ventana discovery-ultra (Roche Diagnostics, Indianapolis, IN), under the conditions detailed in **Supplementary Tables S3**, **S4**, as previously described. Tissue sections were imaged using the Vectra 3 quantitative pathology imaging system (Akoya Biosciences, Marlborough, MA) at 20× magnification, with exposure settings optimized for each Opal fluorophore to avoid signal saturation (e.g., Opal 520, 540, 570, 620, 650, 690). Regions of interest (ROIs) were acquired from at least 50% of the tissue area for macrophage panel and from approximately 100% for the T cell panel, corresponding to 30–60 images per liver biopsy and ensuring substantial tissue representation. Imaging was performed using stamped acquisition areas of 2 × 2 (1,338 μm × 1,000 μm) at a resolution of 0.5 μm (20×) (Saldarriaga et al., 2024). ROIs included both portal tracts and lobular regions, and no additional tissue segmentation was applied. Images and ROIs were selected using automated detection and processed with inForm software (Akoya Biosciences, Marlborough, MA), to generate multi-component TIFF files, which were subsequently analyzed in Visiopharm (Hoersholm, Denmark) using deep learning-based AI algorithms for tissue and nuclear identification, and cell phenotype classification. Nuclei were detected based on DAPI staining using a segmentation procedure that includes a trained neural network algorithm as well as size and shape thresholds to separate overlapping nuclei (typical size range 5–20 µm²). Fluorescent markers were quantified in the corresponding channels within nuclear (e.g., Mac387) or cytoplasmic (e.g., CD163) masks, and cells were classified based on intensity thresholds or machine-learning classifiers trained on representative ROIs. To compare marker expression across patients, cell counts were quantified as the proportion of marker-positive cells relative to the total cell population (DAPI +). Inter- and intra-run reproducibility were managed using a semi-automated staining protocol combined with optimized AI algorithms for quantification, which were validated for each patient sample prior to automated analysis. AI-assisted analysis provides automated, reproducible cell-type quantification within automatically selected ROIs, validated through expert review.

### Statistical analysis

Histological, laboratory data and gene expression analyses were conducted in R (v4.4.3) using the limma package (v3.62.2). Prior to differential expression analysis, we assessed the relationship between RNA quality and sample storage characteristics. A multiple linear regression model was employed to evaluate the impact of storage duration (in years) and storage type (FFPE vs. fresh frozen) on RNA quality using the lm function in R. The storage type was identified as a confounder (β = 69.28, SE = 16.62, p < 0.001) and incorporated as a covariate in the limma design matrix. To further account for both paired and unpaired samples across control, pre, and post groups, within-patient correlations were estimated using duplicateCorrelation. Linear models were then fit with lmFit (block = patient_id) incorporating the consensus correlation, followed by empirical Bayes moderation (eBayes) to stabilize variance estimates. Pairwise contrasts (pre vs control, post vs control, post vs pre) were specified with makeContrasts, and moderated t-statistics and raw p-values were calculated. P-values were adjusted for multiple testing using the Benjamini-Hochberg (B-H) procedure to control the false discovery rate (FDR), allowing inclusion of all samples while accounting for repeated measures. Normality of the cell marker data and other variables was assessed using the Shapiro–Wilk test. Depending on the comparison, paired or unpaired t-tests were applied as appropriate. To account for multiple comparisons (macrophages and T cells phenotypes), raw p-values were adjusted using the Bonferroni method in R, yielding the final adjusted p-values. Associations between pre-DAA gene expression and clinical outcomes were determined using Fisher’s exact test, with odds ratios (OR) >1 indicating positive associations. To assess potential confounding variables affecting outcomes (poor vs. good), we performed multivariable logistic regression in R, adjusting for baseline fibrosis stage, MHAI scores, and HIV status. Boxplots, volcano plots, PCAs, and heatmaps were generated using GraphPad Prism v10.3.0 (GraphPad Software, San Diego, CA, USA).

## Results

### Study design

**Figure 1:**
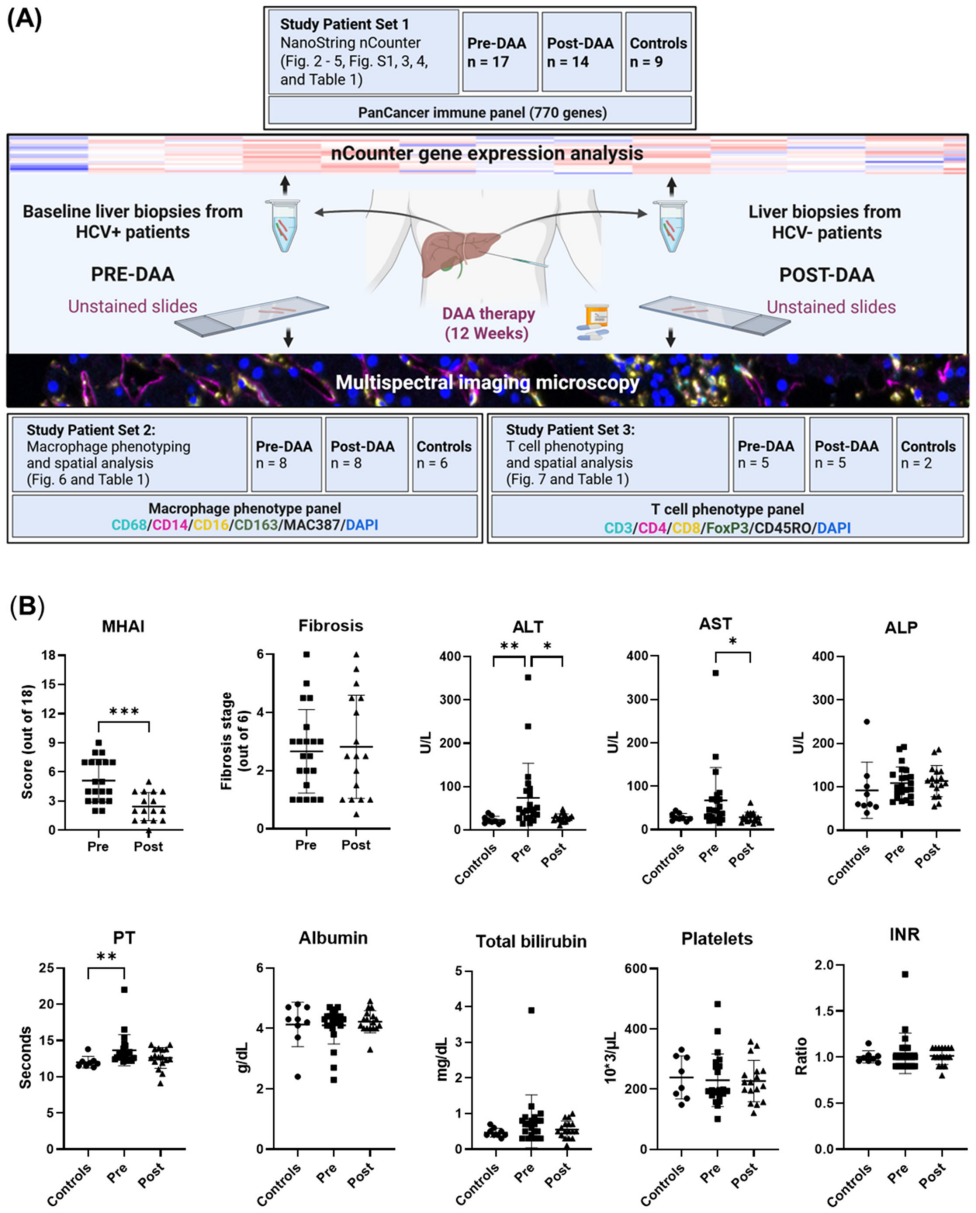
Experimental workflow and comparison of clinical data of patients pre- and post-DAA treatment for viral hepatitis C. **(A)** This study evaluated differences in the hepatic microenvironment of liver biopsies collected from patients with HCV (n = 22: 16 patients had paired pre-and post-treatment biopsies, five had only pre-treatment biopsies, and one had only a post-treatment biopsy, see also **Table 1**) and controls (n = 15; two controls used for T cell phenotyping were also used for macrophage phenotyping analysis) using gene expression and spectral imaging (see also **Supplementary Table S1**). Post-treatment biopsies were collected after SVR. RNA was extracted from fresh liver and FFPE slides for nCounter analysis (Set 1). Spectral imaging was performed on pre- and post-DAA biopsies using macrophage (Set 2) and T cell (Set 3) panels. Multicomponent TIFFs were imported into the Visiopharm digital software platform to generate phenotype profile maps. **(B)** Clinical comparison of patients pre- and post-DAA, and controls. Post-DAA patients showed significantly reduced inflammatory activity (MHAI score) and lower ALT/AST levels. Liver biopsies were graded for inflammatory activity using the MHAI scoring criteria (0-18) and staged for fibrosis using Ishak (1-6) criteria. Histological and laboratory data were analyzed using limma (R/Bioconductor), accounting for paired (pre/post) and unpaired samples with duplicateCorrelation function; linear models with empirical Bayes moderation were used, and p-values were adjusted by the B-H FDR method. ***p < 0.001. Pre, pre-DAA; Post, post-DAA; MHAI, modified hepatitis activity index; ALT, alanine transaminase; AST, aspartate aminotransferase; ALP, alkaline phosphatase; FFPE, formalin-fixed paraffin-embedded; PT, prothrombin time; INR, international normalized ratio.

This study aimed to analyze gene and protein expression changes in pre- and post-DAA treatment to assess immune responses following HCV clearance. Gene and protein signatures were also correlated with inflammatory activity, fibrosis stages, and clinical outcomes. We previously correlated the light microscopy findings with clinical outcomes in 10 patients (Saldarriaga et al., 2021). Here, we expanded the cohort to 22 patients, analyzing nCounter gene expression and multispectral imaging in parallel patient subsets with partial overlap, to evaluate hepatic immune dynamics over time. The experimental workflow is shown in **Figure 1A**. Liver biopsies were collected pre- and post-DAA treatment. RNA from Study Patient Set 1 underwent NanoString nCounter gene expression analysis. Unstained biopsy sections from Study Patient Set 2 were stained with a macrophage panel, whereas slides from Study Patient Set 3 were stained with a T cell panel, acquired with multispectral imaging, and analyzed using Visiopharm. Patients analyzed in the different experiments partially overlap, with some included in both gene expression and macrophage or T cell panels (**Table 1**; **Figure 1A**).

**Table 1:** Demographic and laboratory data for patients with HCV pre- and post-DAA treatment analyzed with nCounter and multiplexing imaging.

Set #	Pt #	Pre/ Post	Age	Sex	F/P (m)	Dur. pre/ Post-Bx	Dur. SVR post-Bx	Ethnicity /Race	HCV Geno-type	HCV VL Log_10_ IU/mL	*** MHAI n/18	Fibrosis n/6	Fat (%)	*** ALT	*** AST	ALP	ALB	Tot BIL	PLT	PT	INR	Outcomes^a^	T cell panel	MAC panel	nCounter	DAA^b^
2/3	1	Pre	51-60	M	68	54	14	NHW	1a	4.64	7	3	1	90	66	93	4.6	0.3	392	14.3	1.1	No Cirrhosis, No HCC	YES	YES	NA	Sofosbuvir/ simeprevir
1/2/3		Post	61-70							SVR12	5	2-3	1	27	22	77	4	0.3	358	12.4	0.9		YES	YES	Post-cold	
2	2	Pre	41-50	F	32	16	9	NHW	1a	5.39	9	2	40	352	361	140	4.1	0.8	157	13.4	1	No Cirrhosis, No HCC	NA	YES	NA	Sofosbuvir /ledipasvir
2		Post	41-50							SVR12	4	2	20	22	16	136	4	0.5	223	12.6	0.9		NA	YES	NA	
2	3	Pre	41-50	M	103	70	17	HW	1a	0.958	3	1	0	82	70	128	4.2	0.3	276	12.7	1	No Cirrhosis, No HCC	NA	YES	NA	Sofosbuvir/ simeprevir
2		Post	51-60							SVR12	2	0-1	0	39	29	108	4.7	0.7	259	12.9	1		NA	YES	NA	
2	4	Pre	51-60	F	142	85	28	NHW	2	0.664	8	6	5	239	168	94	4.3	1	101	15.4	1.2	HCC, Deceased	NA	YES	NA	Sofosbuvir/ ribavirin
2		Post	61-70							SVR12	4	6	60	33	38	78	4	0.9	121	14.4	1.1		NA	YES	NA	
1/2/3	5	Pre	31-40	M	111	20	19	NHAA	1a	2.61	4	1	0	40	29	108	4.4	0.5	322	12.7	0.9	No Cirrhosis, No HCC	YES	YES	Pre-cold	Sofosbuvir/ simeprevir
1/2/3		Post	31-40							SVR12	1	1	0	25	25	114	4.9	0.1	344	12.7	1		YES	YES	Post-cold	
1/2	6	Pre	61-70	M	105	15	6	HW	1a	3.08	2	1	0	28	30	123	4.1	0.7	197	13.3	1	No Cirrhosis, No HCC	NA	YES	Pre-cold	Sofosbuvir/ ledipasvir
1/2		Post	61-70							SVR12	0	1	0	23	33	114	4.3	0.5	198	13.7	1.1		NA	YES	Post-hot	
1/2	7	Pre	51-60	F	113	26	24	NHW	1b	3.32	5	3	10	48	53	138	4.4	0.8	194	12.2	0.9	No Cirrhosis, No HCC	NA	YES	Pre-cold	Sofosbuvir/ simeprevir
1/2		Post	51-60							SVR12	2	1	10	30	25	120	4	0.3	210	13.8	1.1		NA	YES	Post-cold	
1/2/3	8	Pre	51-60	M	128	24	20	NHW	2b	17.2	3	2	15	15	23	99	4	0.5	159	12.6	0.9	No Cirrhosis, No HCC	YES	YES	Pre-cold	Sofosbuvir/ ribavirin
1/2/3		Post	51-60							SVR12	2	1	5	33	24	105	4.5	0.6	200	12.3	0.9		YES	YES	Post-cold	
	9	Pre	51-60	M	84	NA	26	NHW	2b	3.96	NA	NA	NA	35	32	63	4.4	0.6	145	13	1	No Cirrhosis, No HCC	NA	NA	NA	Sofosbuvir/ simeprevir
1		Post	51-60							SVR12	1	1-2	10	48	38	55	4.3	0.9	240	12.7	1		NA	NA	Post-cold	
1	10	Pre Post	41-50 41-50	F	45	21	15	NHAA	1b	2.94	8	4-5	5	122	133	187	4.3	0.8	179	13.2	1	ChCA, Deceased	NA	NA	Pre-hot	Viekira Pak
1										SVR12	3	4	2	10	20	135	4	0.5	247	13.8	1.1		NA	NA	Post-cold	
1	11	Pre Post	51-60	F	56	24	86	H	1a	0.011	4	3	10	27	23	102	2.3	0.3	298	13.9	1.1	No Cirrhosis, No HCC	NA	NA	Pre-hot	Elbasvir/ grazoprevir
1			51-60							SVR12	2	3	5	18	13	104	4.1	0.7	261	13.9	1.1		NA	NA	Post-cold	
1/3	12	Pre	51-60	M	100	34	26	W	1a	0.22	3	2-3	5-10	35	21	69	4.2	0.3	289	12.2	0.9	No Cirrhosis, No HCC	YES	NA	Pre-hot	Sofosbuvir/ ledipasvir
1/3		Post	51-60							SVR12	2	2-3	5-10	25	16	121	4.7	0.5	328	9.1	0.8		YES	NA	Post-hot	
1/3	13	Pre	41-60	M	103	45	31	HW	1a	0.053	5	5	5	51	46	120	4.7	0.7	198	14.6	1.1	Cirrhosis	YES	NA	Pre-hot	Viekira Pak
1/3		Post	51-60							SVR12	2	4-5	0	20	19	141	4.3	0.3	195	10.4	1		YES	NA	Post-cold	+ribavirin
1	14	Pre	61-70	M	54	NA	NA	AA	1a	4.94	7	3	0	21	20	67	3.8	0.4	185	12.1	0.9	Deceased	NA	NA	Pre-hot	No Tx^c^
1	15	Pre	51-60	M	96	NA	NA	AA	1a	2.49	2	1	0	27	44	121	4.1	3.9	257	12.4	0.9	No Cirrhosis, No HCC	NA	NA	Pre-cold	Sofosbuvir/ velpatasvir
1	16	Pre	51-60	F	94	NA	NA	W	1b	0.20	4	2	15	58	70	192	4.3	0.7	274	12.4	0.9	No Cirrhosis, No HCC	NA	NA	Pre-cold	Sofosbuvir/ ledipasvir
1	17	Pre	51-60	M	131	NA	NA	W	2b	2.33	4	2-3	0	44	44	88	4.7	1.2	196	22	1.9	Cirrhosis, Deceased	NA	NA	Pre-cold	Sofosbuvir/ velpatasvir
1	18	Pre	51-60	F	39	NA	NA	W	2b	0.034	3	1-2	0	45	32	78	4.6	0.5	482	12.7	1	No Cirrhosis, No HCC	NA	NA	Pre-cold	Sofosbuvir/ velpatasvir
1	19	Pre	61-70	M	79	68	62	W	3a	ND	7	4-5	0	47	37	74	4.4	0.9	193	12.7	0.9	Cirrhosis, HCC, Deceased	NA	NA	Pre-hot	Sofosbuvir/ daclatasvir /ribavirin
1		Post	61-70							SVR12	3	4-5	0	19	36	61	4.1	0.7	148	12.1	1.1		NA	NA	Post-cold	
1	20	Pre	51-60	F	161	125	17	AA	1a	0.20	4	1	5	95	80	86	3.2	0.3	179	13.3	1	Cirrhosis	NA	NA	Pre-hot	Sofosbuvir/
1		Post	61-70							SVR12	4	5	15	28	38	180	4.2	0.4	223	11.9	1		NA	NA	Post-cold	velpatasvir
1	21	Pre	51-60	M	77	57	9	AA	1a	0.73	7	3	0	107	85	160	4.4	0.9	189	13	1	No Cirrhosis, No HCC	NA	NA	Pre-hot	Glecaprevir/ pibrentasvir
1		Post	51-60							SVR12	2	2-3	1	31	62	97	4.4	1	157	11	1		NA	NA	Post-cold	
1	22	Pre	41-60	M	21	7	5	HW	1b	ND	7	3-4	0	14	15	65	2.7	0.8	193	16.5	1.3	Cirrhosis	NA	NA	Pre-hot	Peg- Ribavirin^d^
1		Post	41-60							SVR12	4	5-6	0	40	28	186	3.3	0.5	152	14.4	1.1		NA	NA	Post-hot	

Set 1: Patients included in the gene expression analysis (nCounter). Set 2: Patients analyzed using multispectral imaging microscopy with the macrophage panel. Set 3: Patients analyzed using multispectral imaging microscopy with the T cell panel. Normal range: ALT, 9‐51 U/L; AST, 13‐40 U/L; ALP, 34-122 U/L; ALB, 3.5-5 g/dL; Tot BIL, 0.1-1.1 mg/dL; PLT,166-358 x1000/µL; PT, 11-13.5 sec; INR, 0.8-1.2

Abbreviations: Pt #, patient number; Pre, pre-treatment; Post, post-treatment; Post^***^, patients with persistent inflammation post-DAA by histopathology (MHAI>2); M, male; F, female; F/P, Follow-up (months); Dur. (Pre/Post Bx), Duration SVR Post-therapy biopsy (months); Dur. SVR Post-Bx, Duration between Pre/Post therapy biopsy (months); H, Hispanic; W, White; AA, African American; NHW, Non-Hispanic white; HW, Hispanic white; NHAA, Non-Hispanic African American; HCV, hepatitis C virus; VL, viral load; SVR12, undetectable HCV RNA in serum 12 weeks after completing antiviral therapy; MHAI, modified hepatic activity index (0-18); ALT, Alanine transaminase; AST, Aspartate aminotransferase; ALP, Alkaline phosphatase; ALB., albumin; Tot BIL, total bilirubin; PLT, Platelets; PT, Prothrombin time;  INR, International Normalized Ratio; HCC, Hepatocellular carcinoma; ChCA, cholangiocarcinoma; MAC, macrophage; DAA, Direct-acting antivirals; NA, not available; ND, not detected.

Clinical parameters were analyzed with limma (R/Bioconductor), accounting for paired and unpaired samples with within-patient correlations. Moderated t-statistics were computed, and p-values were adjusted for multiple testing using the Benjamini-Hochberg method. Asterisk on top of MHAI, ALT, and AST represent significant statistical differences between the groups (***p < 0.001). Superscript a: Patient 4 and 10 succumbed to HCC and ChCA, respectively, while patients 14 and 19 died of cirrhosis-related complications. Patient 13 died of cardiogenic shock. b: All patients completed the duration of the treatment according to the guidelines at the time of treatment except patients 22 and 14. c: Patient did not receive any treatment, d: Treatment was discontinued after 5 weeks due to renal complications.

### Demographics and clinical data

Patient demographics, histological scoring, and clinical data, including viral loads and genotypes, are shown in **Table ​1**. The cohort included 14 males and eight females, with a mean age of 52.45 ± 6.58 years at first biopsy and 54.82 ± 7.82 years post-treatment. Patients were predominantly White (64.29%) and non-Hispanic (77.0%). Body mass index (BMI) remained stable from baseline to post-treatment (28.22 ± 6.09 kg/m^2^ to 29.70 ± 6.97 kg/m^2^, p = 0.52 (**Supplementary Table S1**; **Table 1**). The control group included eight males and seven females (mean age: 53.8 ± 12.9 years). Race distribution was 80.0% White and 20.0% non-White, and ethnicity distribution was 20% Hispanic and 80% non-Hispanic. Although controls, were not matched to study patients, no significant differences in age, sex, race, ethnicity, or BMI were observed when compared with the pre-DAA group (**Supplementary Table S1**). The most prevalent genotype in the pre-DAA treatment group was 1a (55%), followed by 1b (18%) and 2b (18%) (**Table 1**). After treatment, inflammatory activity (MHAI) decreased significantly from 4.81 ± 2.10 to 2.40 ± 1.14, along with AST (67 ± 74 U/L to 28 ± 11.56 U/L) and ALT (74 ± 79.64 U/L to 28 ± 11.6 U/L) (p < 0.001). Fibrosis stage and major liver function and coagulation markers (ALP, PT, albumin, total bilirubin, platelets, INR) showed no significant change (**Figure 1B**).

### Gene expressions change pre- and post-DAA treatment

**Figure 2:**
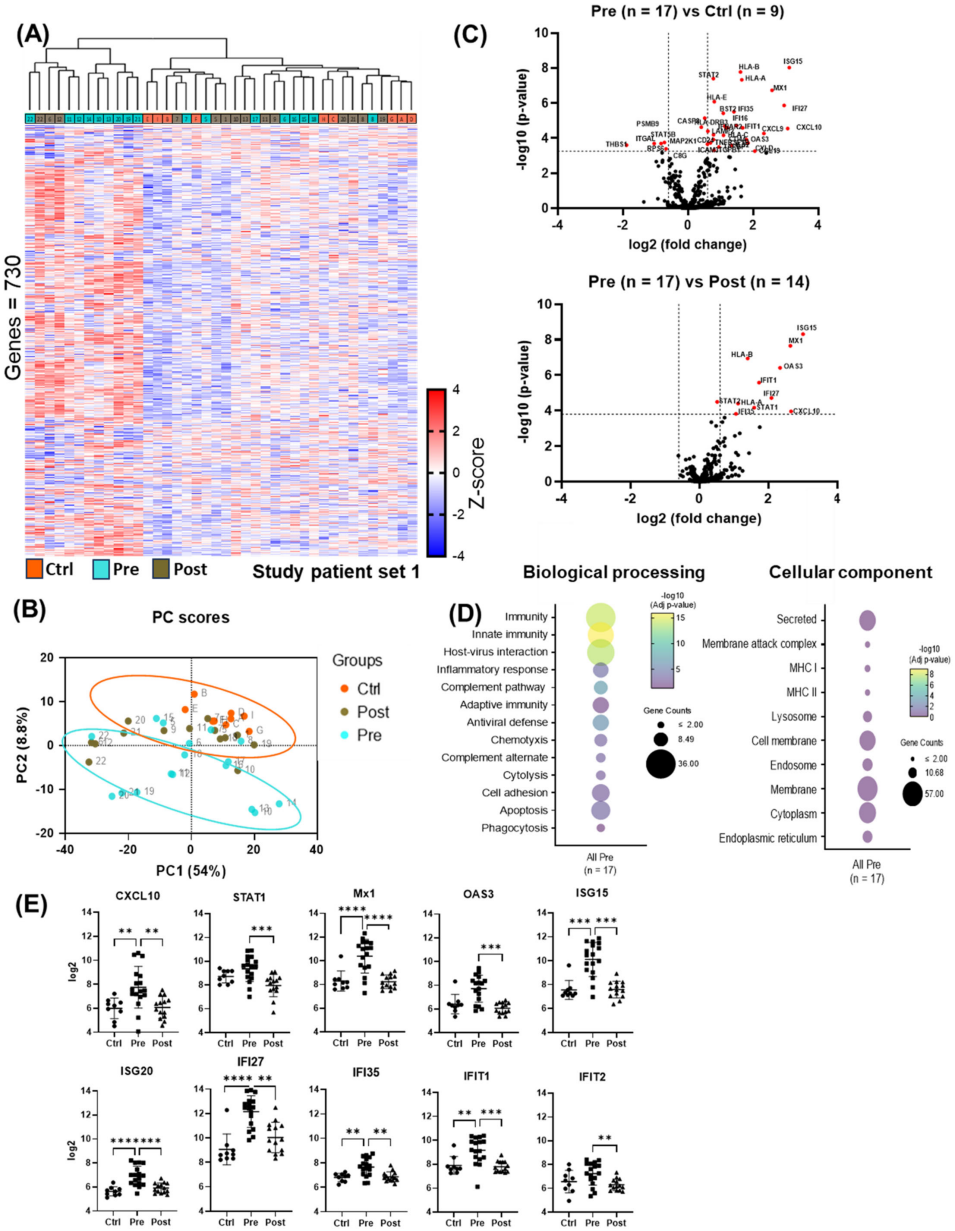
nCounter analysis showed reduced antiviral and pro-inflammatory gene expression post-DAA therapy. **(A)** Cluster analysis of liver biopsy gene expression (controls (n = 9), pre-DAA (n = 17), and post-DAA (n = 14) patients). The heat map displays two primary clusters that predominantly represent pre-DAA samples and post-DAA/control samples. All clustering and label assignments were performed without reference to patient outcomes, ensuring that the definitions were unbiased and purely based on expression data. The dendrogram is presented as an exploratory tool to illustrate the structure of the data without overinterpreting the statistical robustness of each branch. **(B)** PCA of log2-transformed data confirmed this clustering. The colors represent the predefined experimental groups (ctrl, orange; pre-, light green; post-, brown), which were determined by study design and not based on *post hoc* unsupervised labels. **(C)** Volcano plots comparing pre-DAA vs. controls and pre- vs. post-DAA show antiviral/pro-inflammatory genes upregulated pre-DAA and returning to baseline post-treatment. **(D)** Differential gene expression obtained from comparing pre-DAA versus control groups was used to determine enriched pathways using DAVID software and the Reactome database. The y-axis represents the top enriched pathways; the dot color represents the log10 adjusted p-value, with yellow indicating higher significance (–log10 > 2 = ≤0.01). **(E)** Scatter plots show significant post-DAA downregulation of antiviral and type I IFN-induced genes, reaching control levels. Volcano plots show log2 fold change versus –log10 adjusted p-value (Benjamini–Yekutieli), with horizontal dashed lines indicating –log10 BY-adjusted p < 0.05 and vertical dashed lines representing log2 fold change < –0.6 or > 0.6. Gene comparisons were analyzed using limma (R/Bioconductor), accounting for paired (pre/post) and unpaired samples with duplicateCorrelation function; linear models with empirical Bayes moderation were used, and p-values were adjusted by the B-H FDR method. *p < 0.05; **p < 0.01; ***p < 0.001; ****p < 0.0001. Ctrl, Controls; Pre, pre-DAA; Post, post-DAA; PCA, principal component analysis.

Unsupervised Hierarchical clustering of gene expression data (**Figure 2A**) revealed two distinct clusters: one with low (“cold”) overall gene expression profile [controls (eight out of eight), most post-DAA (eleven out of fourteen), half pre-DAA (eight out of seventeen)] and another with high (“hot”) gene expression profile [half pre-DAA (nine out of seventeen), a few post-DAA (three out of fourteen)]. Principal component analysis (PCA, **Figure 2B**), using all genes, confirmed the same separation, with cluster assignments from **Figure 2A** used to outline groups. The reproducibility of the clustering was supported by the consistent identification of the same two groups using both hierarchical clustering and PCA.

Compared to controls, pre-DAA treatment patients exhibited upregulation of inflammatory and antiviral genes (*STAT2, CXCL10, CXCL9, IFNAR2, ISG15, OAS3, MX1, IFI27, IFI35*), highlighting an active immune response to chronic HCV infection. These expression levels declined post-DAA treatment, aligning with controls and indicating resolution of the antiviral immune response (**Figure 2C**; **Supplementary Tables S5**, **S6**), consistent with previous reports (Boldanova et al., 2017). Pathway analysis revealed the enrichment of immune-related and host-virus interaction pathways pre-DAA, with a significant decrease observed post-DAA, returning to levels comparable to controls (**Figures 2D, E**). Inflammatory (*CXCL10, STAT1*) and interferon-induced antiviral genes (*MX1, OAS3, ISG15, ISG20, IFI27, IFI35, IFIT1, IFIT2*) were significantly downregulated post-DAA (p < 0.05), consistent with the resolution of the antiviral immune response as previously reported (Burchill et al., 2021). Marked heterogeneity was observed in log2 expression levels across patients pre- and post-DAA (**Figure 2E**).

**Figure 3:**
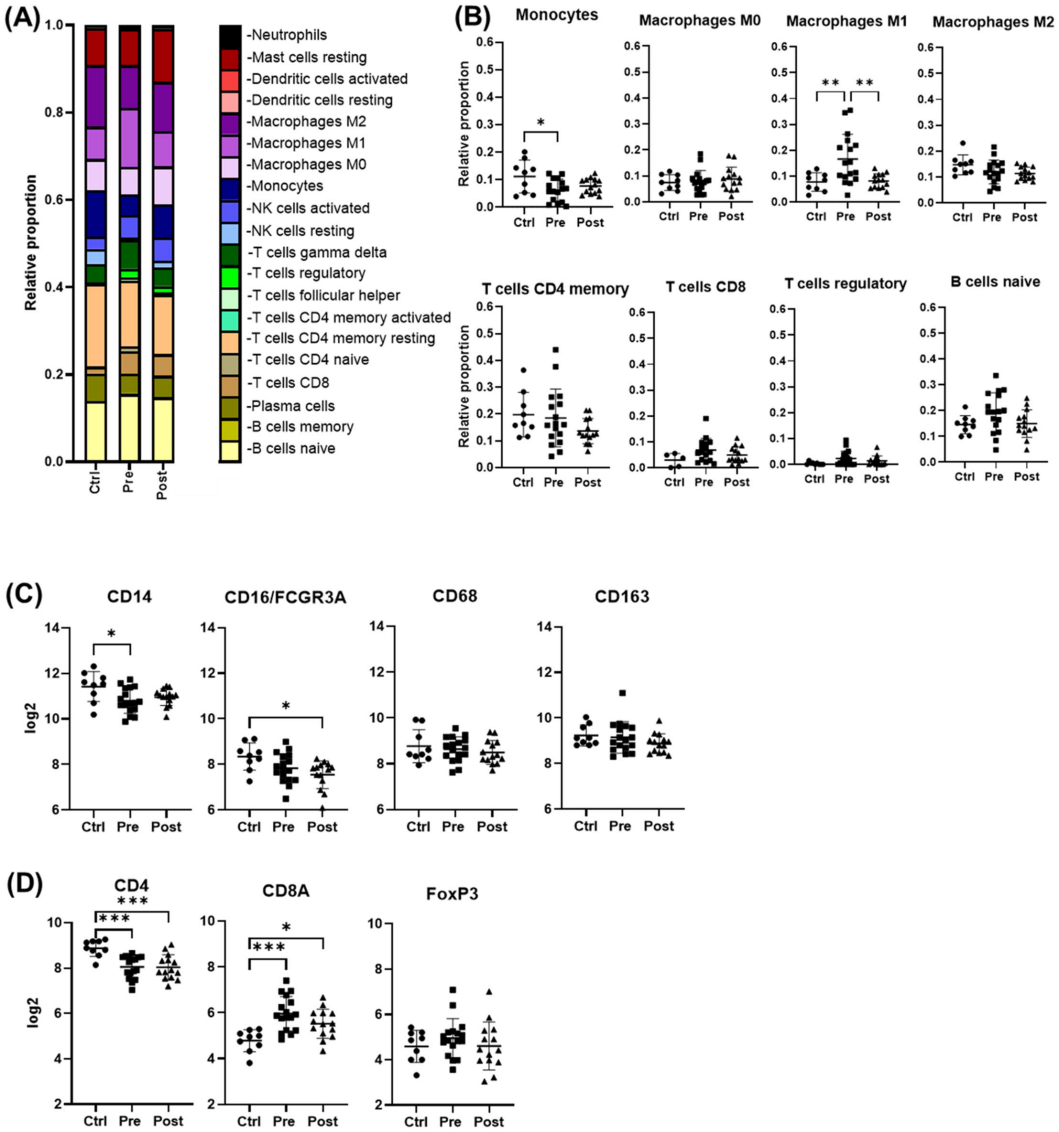
Cell deconvolution analysis reveals distinct immune cell dynamics post-DAA therapy. Gene expression was analyzed using CIBERSORT (Chen et al., 2018). We applied this tool, which uses a reference dataset of gene expression signatures from known cell types, to estimate the relative proportions of different immune cell types in liver biopsies from controls (n = 9) compared with those from patients pre- (n = 17) and post-DAA treatment (n = 14). **(A, B)** CIBERSORT showed individual variation in macrophage and T cell populations. M1 macrophages significantly decreased post-DAA, resembling controls. Pre-DAA enriched M1 and T cell genes are shown in **Supplementary Figure S1A**. **(C, D)** Box plots compare log2 gene expression of resident, pro-inflammatory, and anti-inflammatory macrophages, and T cell markers among the groups. Gene expression was analyzed using limma (R/Bioconductor), accounting for paired (pre/post) and unpaired samples with duplicateCorrelation function; linear models with empirical Bayes moderation were used, and p-values were adjusted by the B-H FDR method. *p < 0.05; **p < 0.01. Ctrl, Controls; Pre, pre-DAA; Post, post-DAA.

Cell deconvolution analysis revealed that, among the immune cells shaping the liver landscape in the patient groups, M1 macrophages were elevated pre-treatment (p < 0.05) but declined significantly post-DAA (p < 0.01), consistent with reduced hepatic inflammation. T cells showed a trend toward increased gene expression pre-DAA compared to controls, with CD8+ T cells showing a significant (p<0.05) increase pre-DAA that remained unchanged post-DAA (**Figures 3A, B**). Unexpectedly, *CD14* and *CD16* gene expression were highest in controls, with no significant differences between pre- and post-treatment groups (**Figure 3C**). Notably, *CD4* expression was significantly higher in the controls compared to pre- and post-treatment groups, while *CD8A* a marker of cytotoxic CD8+ T cells, was upregulated (p<0.05) in the pre-DAA group compared to controls (**Figure 3D**; **Supplementary Figure S1**). These gene expression findings motivated a complementary analysis of protein expression in a separate subset of patients (see multispectral imaging section).

### Gene expression revealed distinct sub-clusters in HCV patients pre- and post-DAA

**Figure 4:**
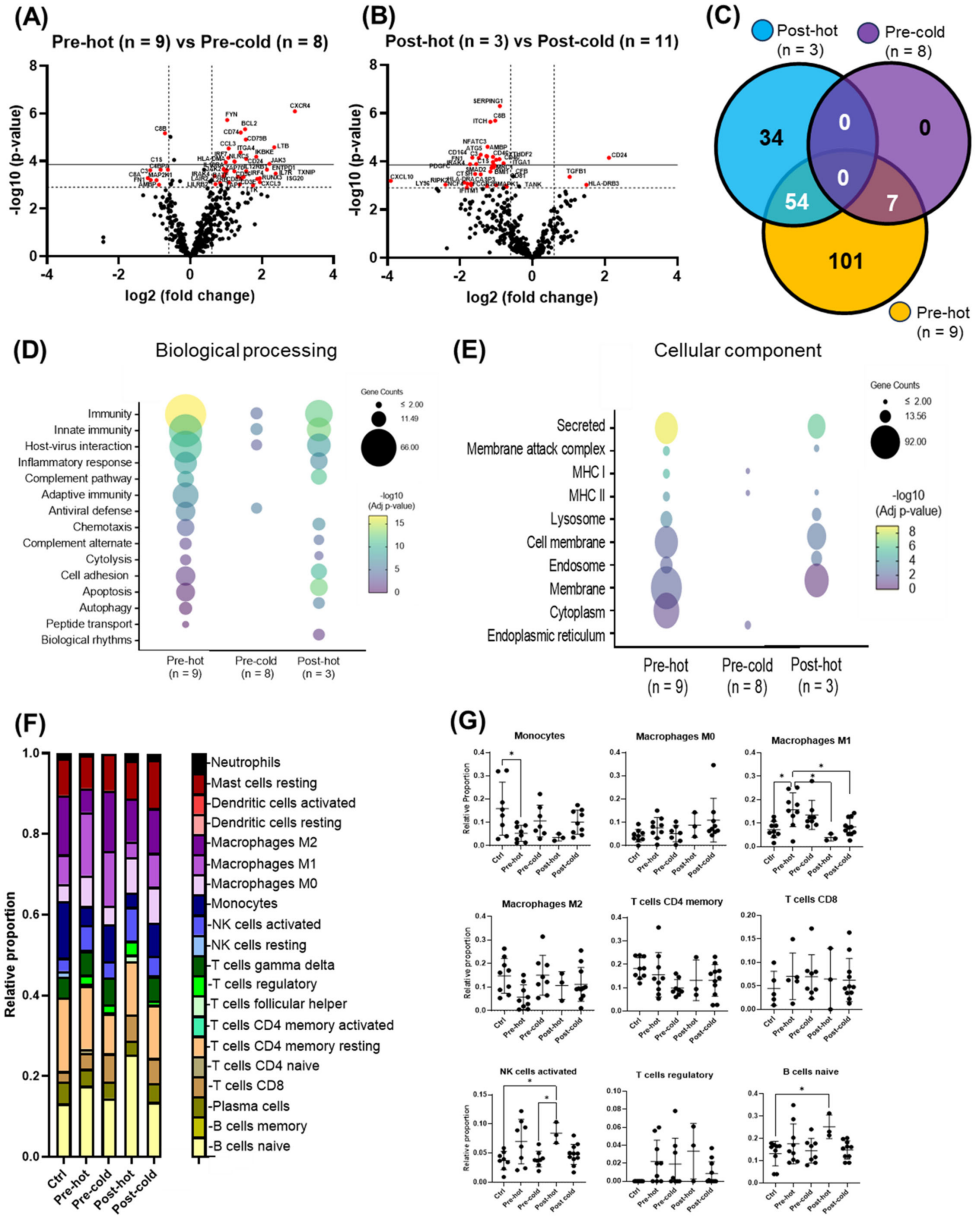
Gene expression patterns within the pre- and post-DAA groups revealed distinct individual responses to HCV infection and DAA therapy. In patients infected with HCV and then treated with DAA (see **Figure 2A**), we identified different subclusters in the heat map: pre-hot (high expression pre-DAA vs. controls), pre-cold (low expression pre-DAA vs. controls), post-hot (high expression post-DAA, like pre-hot), and post-cold (low expression post-DAA, like controls). **(A, B)** Volcano plots compare gene expression in pre-hot vs. pre-cold and post-hot vs. post-cold groups. **(C)** The Venn diagram illustrates significantly expressed genes across pre-cold, pre-hot, and post-hot patients and their overlap. Of the 197 differentially expressed genes (the pre-hot expressing 162 genes), 54 genes overlap between the post-hot and pre-hot groups, while seven genes overlap between the pre-cold and pre-hot groups. In addition, the pre-cold showed a gene expression pattern like the controls, as it had the fewest significant genes (7 genes) compared to the other groups. **(D, E)** Differential gene expression obtained from comparing pre-hot, pre-cold, and post-hot groups versus liver controls (see **Supplementary Figures S3**, **S4**; **Supplementary Table S8**, **S10**, **S12**) was used to determine enriched pathways using DAVID software and the Reactome database. The y-axis represents the top enriched pathways. The color of the dots represents the -Log10 of the adjusted p-value obtained from the enrichment analysis. –Log10 > 2 (adjusted p-value= ≤0.01) indicates significant enrichment (yellow dots represent higher significance). Compared to the pre-hot group, the post-hot group was characterized by activating persistent immune and inflammatory response pathways without activating antiviral genes and MHC I pathways. **(F, G)** Gene expression was analyzed using CIBERSORT to estimate the relative proportions of different immune cell types in controls and pre- and post-DAA treatment groups (pre-hot, pre-cold, post-hot, and post-cold). Pre-hot patients showed increased proportions of M1 macrophages, activated NK cells, and naïve B cells. Specific macrophage (M1 and M2), T and B cells, and NK cells genes enriched in each group are shown in **Supplementary Figures S1A, B**, with corresponding laboratory values in **Supplementary Figure S2**. Additional volcano plots and contributing genes are in **Supplementary Figure S3** and **Supplementary Tables S7**-**S13**. Volcano plots show log2 fold change versus –log10 adjusted *p*-value (Benjamini–Yekutieli), with horizontal dashed lines indicating –log10 BY-adjusted *p* < 0.05 and vertical dashed lines representing log2 fold change < –0.6 or > 0.6. Gene expression was analyzed using limma (R/Bioconductor), accounting for paired (pre/post) and unpaired samples with duplicateCorrelation function; linear models with empirical Bayes moderation were used, and p-values were adjusted by the B-H FDR method. *p < 0.05; **p < 0.01; ***p < 0.001; ****p < 0.0001.

Within the pre-DAA group, two subgroups emerged: pre-hot (high inflammatory gene expression, n = 9) and pre-cold (low gene expression, n = 8) (**Figure 2A**). Pre-hot patients exhibited increased inflammatory (*CXCR4, BCL2, FYN, NLRC5, LTB, CCL3, IKBKE, IRF7, JAK3, TNFAIP3, CXCL9*) and cancer progression gene markers (i.e., *CXCR4, BCL2, FYN*) (Lin et al., 2014; Hosseini-Khah et al., 2021; Huang et al., 2022) (**Figures 4A, C–E**; **Supplementary Figures S2**, **S3A–F**, **S4A–D**; **Supplementary Tables S7**-**S9**). In contrast, pre-cold patients retained antiviral and innate immune responses without marked inflammation, representing a distinct immunological profile (**Figures 4A, C–E**; **Supplementary Figures S2**-**4**, **Supplementary Table S10**). No statistically significant differences were observed in histopathological features (MHAI and fibrosis) or laboratory values when compared Pre-hot with the pre-cold group (**Supplementary Figure S2**). Similarly, a post-DAA group formed two subclusters: post-hot (n = 3) clustering along with the pre-hot subcluster, and post-cold (n = 11) near the control group (**Figure 2B**; **Supplementary Figures S3B, D**). Post-hot patients exhibited persistent immune regulation and activation of oncogenic pathways (*TGFβ1* and *CD24*) with downregulation of complement (*C8B, CFB, C1S, SERPING1, C3, CD46, CD81, CD40*) and fibroblast proliferation genes (*PDGFC, BMI1, FN1, SMAD2*), implying potential ongoing tissue remodeling (**Figures 4B–E**; **Supplementary Figure S3**; **Supplementary Tables S11**, **S12**). Cell deconvolution analysis further showed that M1 macrophages were significantly elevated in the pre-hot group compared to the pre-cold group (p < 0.01) and decreased following treatment (**Figure 4B**). Additionally, activated NK cell and naive B cell proportions were significantly increased pre-DAA (p < 0.01) compared to controls, suggesting continued immune engagement. Post-cold patients, in contrast, closely resembled controls, indicating immune normalization (**Figures 4F, G**; **Supplementary Figures S1B**, **S2**, **S3**; **Supplementary Table S13**).

**Figure 5:**
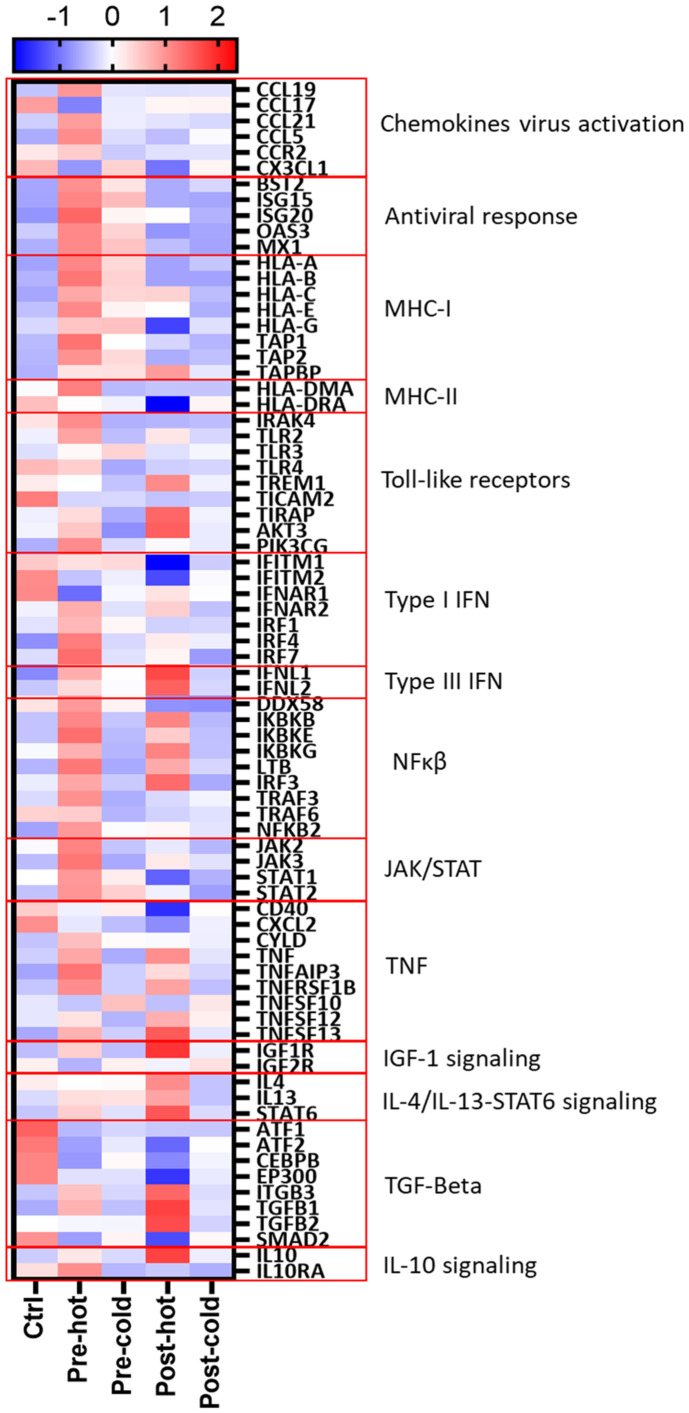
Gene expression patterns reveal distinct inflammatory and fibrogenic pathways despite DAA therapy. Differentially expressed genes (**Supplementary Tables S8**–**S13**; pre-hot, pre-cold, post-hot, and post-cold groups compared to controls) were analyzed using STRING network clustering (MCL inflation = 5) and visualized in a heatmap using the average Z-score value (intensity range: ± 2) for each group. Red boxes highlight key pathway clusters. Antiviral response and MHC-I markers were only upregulated in pre-DAA groups (pre-hot and pre-cold). Inflammation in both pre-DAA groups was linked to type I interferon and TNF/NFκB signaling, with lower expression in the pre-cold group. The post-hot group showed upregulation of inflammatory pathways, including TNF/NF-κB, and anti-inflammatory and profibrotic pathways, such as TGF-β1/2 and IL-4/IL-13/STAT6. pre-hot, high gene expression pre-DAA; pre-cold, low gene expression pre-DAA; post-hot, high gene expression post-DAA; post-cold, low gene expression post-DAA; Ctrl, controls.

The heatmap in **Figure 5** summarizes activated relevant pathways in each subcluster. The pre-hot group showed stronger gene upregulation than the pre-cold group, enriched in type I/III interferon and antiviral response pathways. After achieving SVR, post-DAA patients showed reduced innate immune and antiviral gene expression. Still, post-hot patients retained mixed activation of inflammatory (*TNF, TLRs, NFκB*) and anti-inflammatory/profibrotic (*TGFβ, IL-4, IL-13, STAT6*) pathways.

### Anti-inflammatory macrophages are the predominant phenotype in the hepatic microenvironment after DAA therapy

**Figure 6:**
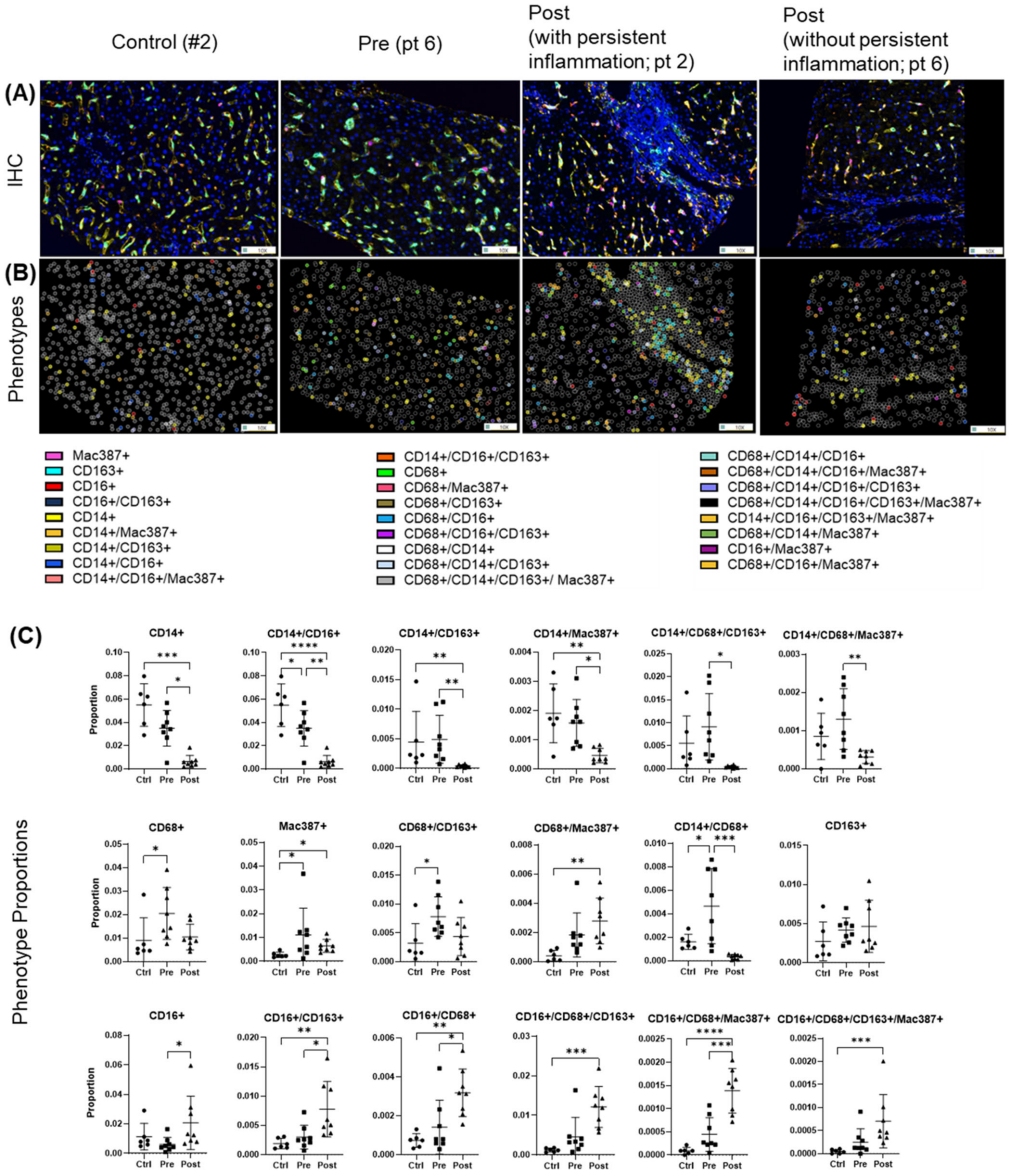
Macrophage phenotypes in liver biopsies pre- and post-DAA therapy reveal a dominant anti-inflammatory response post-treatment. Biopsies were stained with the macrophage multiplex panel (CD68, CD14, CD16, CD163, Mac387, and DAPI), and images from the stained slides were acquired using the Vectra 3 automated quantitative pathology imaging system. Multicomponent TIFF images were analyzed with Visiopharm using optimized custom AI applications to determine the type and number of macrophage phenotypes in the hepatic microenvironment (controls, n = 6; patients with HCV pre-DAA, n = 8; and post-DAA, n = 8). **(A)** Representative multiplex images and **(B)** phenotypes from controls, a pre-DAA patient, and two post-DAA patients (with: MHAI > 2 and without: MHAI < 1, persistent inflammation). Each colored dot represents a unique cellular phenotype, and gray dots represent negative cells for all the markers evaluated. **(C)** Scatter plots were used to compare the proportions of specific cell populations across control, pre-DAA, and post-DAA groups. Control patients exhibited a higher abundance of CD14+ cells, which decreased with HCV infection. Pre-DAA patients showed an increase in inflammatory macrophages (CD68+, CD14+, Mac387+, CD68+/CD14+) compared to controls, which generally diminished after DAA treatment, except in one patient (pt 2), who exhibited persistent inflammation. In contrast, post-treatment samples showed increased anti-inflammatory macrophages (CD16+ and CD16+/CD163+) populations. Images were acquired at 20X. Statistical comparisons were performed using paired or unpaired t-tests, with p-values adjusted for multiple comparisons by the Bonferroni method. *p < 0.05; **p < 0.01; ***p < 0.001, ****p < 0.001.

Multiplex imaging provided insight into hepatic macrophage population shifts. CD14+ macrophages, the predominant subtype in controls, along with their subpopulations (e.g., CD14+/CD16+, CD14+/CD163+, CD14+/Mac387+) were significantly decreased during chronic infection (pre-DAA) and declined further following DAA treatment (p < 0.05) (**Figures 6A–C**) (Saldarriaga et al., 2020; Saldarriaga et al., 2024). Resident (CD68+ and CD68+/CD163+) increased pre-DAA (p < 0.05), and decreasing considerably post-DAA, suggesting a resolution of inflammation following viral clearance. Anti-inflammatory macrophages CD16+ and their subpopulations (e.g., CD16+/CD163+, CD16+/CD68+) significantly increased post-treatment (p < 0.05), indicating a shift toward immune resolution and tissue remodeling (**Figures 6A–C**). These results are consistent with the gene expression analysis, which indicated a significant reduction in inflammatory macrophages (M1) following treatment (**Figures 3B**).

### Spectral imaging revealed significant heterogeneity and diverse hepatic T cell populations in both pre- and post-DAA groups

**Figure 7:**
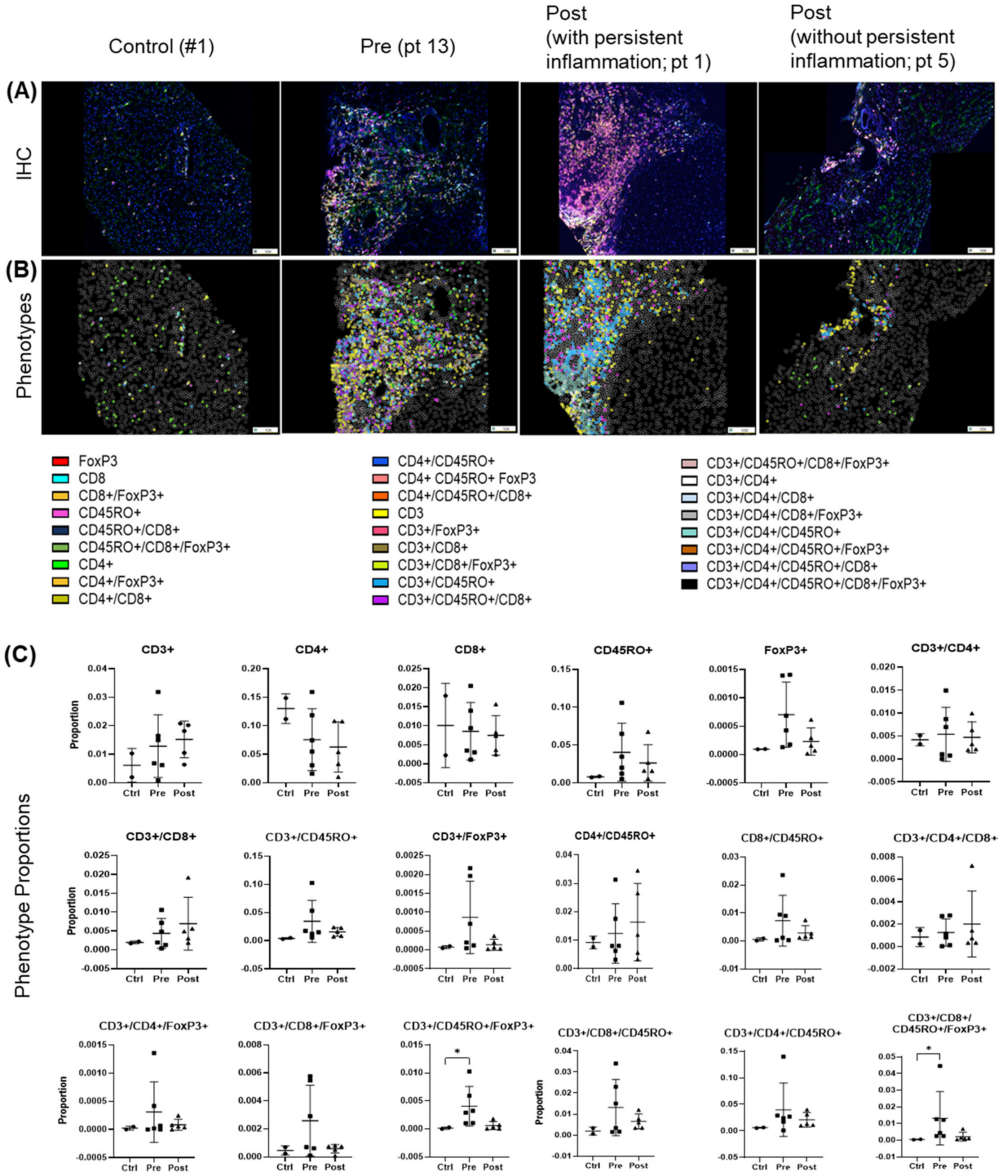
T cell phenotypes in liver biopsies pre- and post-DAA therapy show individual heterogeneity. Biopsies were stained with a multiplex T cell panel (CD3+, CD4+, CD8+, CD45RO+, FoxP3+, and DAPI), and images from the stained slides were acquired using the Vectra 3 automated quantitative pathology imaging system. Multicomponent TIFF images were analyzed with Visiopharm using optimized custom AI applications to determine the type and number of T cell phenotypes in the hepatic microenvironment (controls, n = 2; patients with HCV pre-DAA, n = 5; and post-DAA, n = 5). **(A, B)** Representative multiplex images from controls, a pre-DAA patient, and two post-DAA patients (with: MHAI > 2 and without: MHAI < 1, persistent inflammation). Each colored dot represents a unique cellular phenotype, and gray dots represent negative cells for all the markers evaluated. **(C)** Scatter plots were used to compare the proportions of specific T cell populations across pre-DAA and post-DAA groups. Individual heterogeneity was evident in the pre- and post-DAA groups, with a predominant mixed lymphocytic infiltrate (helper/CD4+, cytotoxic/CD8+, and memory/CD45RO+) before treatment and a mixed memory phenotype (CD3+, CD45RO+, CD3+/CD45RO+, CD4+/CD45RO+, CD3+/CD4+/CD45RO+) post-treatment in patients with persistent inflammation. MHAI, modified hepatitis activity index; AI, artificial intelligence. Statistical comparisons were performed using paired t-tests, with p-values adjusted for multiple comparisons by the Bonferroni method. Controls (n = 2, shown for descriptive purposes only; not included in statistical analysis).

Before treatment, biopsies revealed largely heterogeneous distributions of analyzed T cell populations and their subpopulations across pre- and post-treatment groups. Several populations and subsets, including CD3+, FoxP3+, CD4+/CD45R0+ were modestly elevated in the pre-treatment group, but these trends were not statistically significant and were not different post-treatment (**Figures 7A–C**). Importantly, four out of five patients analyzed by spectral imaging (**Table 1**, subset 3) had persistent hepatic inflammation by histology, consistent with the lack of measurable recovery of T cell subsets. Inter-individual variability and the small sample size likely limited detection, and multiple testing adjustments across T cell phenotypes yielded no significant differences. Persistent portal inflammation (MHAI > 2) was observed in 14 of 17 patients (82.3%, 95% CI: 64.2–100%) post-DAA, indicating residual or ongoing lymphocytic infiltration (**Table 1**). Patient 1, for instance, displayed post-treatment inflammation (MHAI > 2), with increased proportions of single-positive CD3+ and CD45RO+ cells, as well as double-positive CD3+/CD45RO+ memory T cells within the liver (**Figure 7A**). Controls (n = 2) were shown for descriptive purposes only and not included in statistical analysis. These findings highlight the persistence of hepatic inflammation in treated HCV patients, underscoring that the immune microenvironment remains variably activated across individuals despite viral clearance.

Consistent with our spectral imaging results, T cell populations (CD8+) and specific genes (*CD8A*) remain elevated post-treatment (**Figures 3A–C**). In agreement with previous reports of persistent portal tract inflammation (Welsch et al., 2017; Putra et al., 2018; Hsieh et al., 2021; Saldarriaga et al., 2021) our analysis of T cell-related gene and marker expression before and after treatment indicates that lymphocytic persistence within the liver can occur despite successful viral clearance. Similarly, *CD4* gene expression was reduced pre-DAA, likely reflecting clonal exhaustion and deletion of HCV-specific CD4 T cells, and did not significantly recover post-SVR (**Figure 3D**). Although HIV coinfection in some patients may potentially influence *CD4* expression (Gazzola et al., 2009; Corbeau and Reynes, 2011; Young et al., 2012), no significant differences were observed between patients with (pt 2-3, 5-13, 15) and without (pt 1, 4, 14, 16-22) HIV coinfection (**Supplementary Figure S5A**).

### Gene expression and histopathology before DAA treatment and their associations with clinical outcomes

Two pre-DAA subclusters based on gene expression were identified as mentioned above: the pre-hot subcluster (nine out of 17, 52.9%) with enriched gene expression and the pre-cold subcluster (eight out of 17, 47.1%) with low gene expression (**Figures 2**, **4**; **Supplementary Tables S7**–**S10**). Although the pre-hot subcluster exhibited a trend toward higher MHAI scores, fibrosis stage, and ALT and AST levels, no statistically significant differences were observed between the pre-hot and pre-cold subclusters in these or other clinical parameters (**Supplementary Figure S2**; **Table 1**).

**Figure 8:**
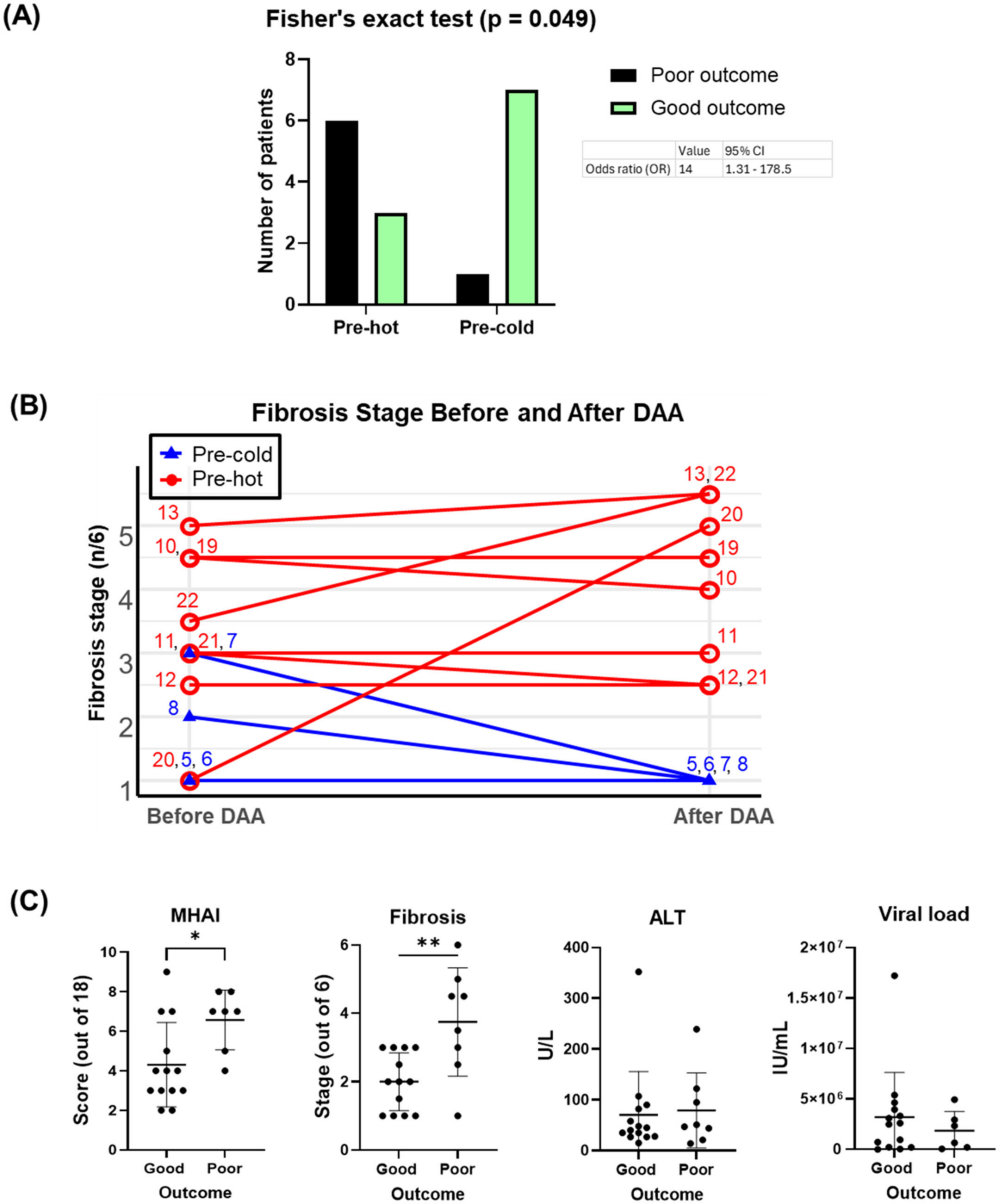
Associations of pre-DAA gene expression profile (Pre-hot and Pre-cold) and baseline histopathology (MHAI, Fibrosis) with clinical outcomes. **(A)** Adverse outcomes occurred more frequently in the pre-hot (seven out of nine, 77.7%) compared to the pre-cold group (one out of eight, 12.5%). Pre-hot gene expression was associated with higher odds (OR: 8) of adverse outcomes although this association was not significant after adjustment for baseline factors such as MHAI and fibrosis. **(B)** Twelve patients with paired liver biopsy samples (pre- and post-treatment) and gene expression profiling (pre-hot and pre-cold) were analyzed. Increased gene expression (pre-hot; pt 10-13, 19-22) before treatment did not improve their fibrosis post-DAA, while it consistently remained low in the pre-cold group (pt 5-8). **(C)** Patients with adverse (poor) clinical outcomes had significantly higher MHAI scores and fibrosis stages at baseline compared to patients with favorable (good) outcomes, but neither variable was a statistically significant predictor of outcomes in regression analyses. Abbreviations: MHAI, modified hepatitis activity index. The Fisher exact test assessed gene expression-outcome association (OR >1 = positive association) To assess potential confounding variables affecting outcomes (poor vs. good), we performed multivariable logistic regression in R, adjusting for baseline fibrosis stage, MHAI scores, and HIV status. Group differences were analyzed using unpaired t-test or the Mann-Whitney test. *p < 0.05; **p < 0.01; ***p < 0.001.

Using Fisher’s exact test, we observed a significant positive association between the pre-hot gene expression profile and adverse outcomes (OR = 14.0, 95% CI: 1.31-178.5; *p* = 0.049) that included cirrhosis, HCC, or liver failure-related death. After adjustment for baseline fibrosis stage, MHAI, and HIV infection status, the pre-hot group remained associated with higher odds of poor outcomes (adjusted OR = 8.04, 95% CI: 0.18-2123.07), although this association was no longer statistically significant (p = 0.328) (**Figure 8A**). Among pre-hot patients, 44.4% (four out of nine) developed cirrhosis, and 33.3% (three out of nine) died from decompensated cirrhosis, compared to only one adverse outcome, 12.5% (one out of eight) in the pre-cold group (**Table 1**).

Among the 16 patients with paired liver biopsies, the fibrosis stage either worsened or remained similar in the pre-hot subcluster. At the same time, fibrosis consistently decreased or remained low in the pre-cold group after DAA treatment (**Figure 8B**). Patients with adverse outcomes had significantly higher MHAI scores (p < 0.05) and fibrosis stages (p < 0.01) pre- treatment than those with favorable outcomes, although ALT levels and viral load did not differ significantly (**Figure 8C**, **Table 1**). To further explore predictive value, we assessed whether MHAI scores and fibrosis stages at baseline could predict outcomes. Each unit increase in MHAI score was associated with nearly double the odds of poor outcomes (OR = 1.90, 95% CI: 0.72-6.34), although this did not reach statistical significance (p = 0.215). Similarly, higher fibrosis stages showed a trend toward poor outcomes (OR = 1.65, 95% CI: 0.44-9.97) but were not statistically significant predictors (p = 0.488), likely reflecting the limited sample size and wide variability.

## Discussion

While HCV is now curable in most individuals (Oru et al., 2021), the risk of liver-related complications remains, particularly in patients with advanced fibrosis or cirrhosis (Saldarriaga et al., 2021). Persistent lymphocytic portal tract inflammation post-treatment has been noted by us and others (Putra et al., 2018; Hsieh et al., 2021; Saldarriaga et al., 2021), raising questions about its clinical implications. Macrophages and T cells play a crucial role in the immune response to HCV, but chronic infection impairs macrophage activation and leads to T cell dysfunction (Tu et al., 2010; Ahmed et al., 2019; Vranjkovic et al., 2019; Zhao et al., 2022). While fibrosis progression is more strongly associated with host factors such as sex, age, and alcohol consumption than with inflammatory activity or viral load (Poynard et al., 1997; Fattovich et al., 2004), the role of immune cell dynamics, particularly post-treatment, remains poorly understood. To address this, we analyzed liver biopsies taken before and after DAA therapy, compared them to those of uninfected controls, and correlated the findings with clinical outcomes.

All patients achieved SVR, with normalized transaminases and reduced inflammation per MHAI scoring. However, unlike other studies, we did not observe a significant reduction in fibrosis after viral clearance (**Figure 1**; **Table 1**) (D'Ambrosio et al., 2012; Bility et al., 2016). HCV infection triggered a potent interferon (IFN)-mediated immune response, which declined following DAA treatment in most patients, reaching levels comparable to those of uninfected controls (**Figure 2E**; **Supplementary Figure S3D**). DAAs are known to suppress ISGs and antiviral pathways in hepatic immune cells (Boldanova et al., 2017; Holmes et al., 2019; Burchill et al., 2021; Listopad et al., 2022; Cui et al., 2024), whether this immunologic shift affects long-term liver health remains uncertain. Unlike IFN-based therapies, DAAs achieve better normalization of these immune responses (Hou et al., 2014).

We observed a significant decrease in inflammatory macrophages (M1-like) following treatment (**Figures 3**, **6**). HCV drove the upregulation of M1-associated markers (e.g., *CD80, CCL5, CXCL9*), which declined after treatment (**Figure 3B**; **Supplementary Figure S1A**). HCV infection elicits dynamic changes in hepatic myeloid cell subsets, particularly CD14+ and CD16+ macrophages, contributing to the entire pool of ISG and MHC-II genes expressed during infection (Cui et al., 2024). Protein analysis in the imaging subset supported a shift from pro-inflammatory to anti-inflammatory macrophage phenotypes, as CD14+ cells declined while CD16+ cells predominated post-SVR (**Figure 6C**). The persistence of *CD16*-expressing subsets, often at higher levels than in uninfected controls, aligns with reports of an expanding *CD16* population after DAAs (Cui et al., 2024). Spectral imaging revealed a significant decrease in CD14+/CD163+ macrophages following DAA and an increase in CD16+/CD163+ subsets (**Figure 6C**), highlighting the advantages of assessing tissue protein expression.

Two distinct pre-treatment inflammatory profiles were identified by gene expression analysis: a “pre-hot” subcluster with high expression of pro-inflammatory (*NLRP3, TLR2-4, NFκB, Jak2-3/STAT1-2, TNF*) and antiviral (*ISG15/20, MX1, OAS3*) genes, as well as type I/III IFNs (*IFITM1-2, IFNAR1-2, IRF1,4, IFNL1-2*) and antigen presentation markers (*HLA-C, TAP1-2, HLA-DMA, HLA-DRA*); and a “pre-cold” subcluster with lower-level expression (**Figures 2**, **4**; **Supplementary Tables S7**–**S10**). While host IFN-λ3/4 gene variants influence ISG expression, HCV pathogenesis, and response to treatment (Terczyńska-Dyla et al., 2014; Salum et al., 2020), we did not assess genetic variations in our cohort. Notably, three patients post-hot maintained high post-SVR expression of type I IFNs (*IFNAR1/2, IRF4*), type III IFNs (*IFNL1-2*), and a mixed inflammatory and anti-inflammatory/profibrotic gene signature (*TLRs, NFκB, TNF, TGFβ, IL4/IL13, IL10*) (**Figures 4B–E**, **5**). Although post-hot patients clustered with the pre-hot patients, their gene expression profile was distinct from both pre-hot and post-cold patients, and, since this pattern was observed in only a small subgroup, this finding should be considered preliminary and hypothesis-generating.

T cell dynamics further contributed to pre- and post-treatment hepatic immune heterogeneity. Restoration of cytotoxic and exhausted CD8 T cells after HCV treatment has been documented (Martin et al., 2014; Osuch et al., 2020), whereas some systemic populations, such as mucosal-associated invariant T cells (MAIT), remain dysfunctional (Hengst et al., 2016). In the gene expression subset, *CD4* expression was reduced in chronic HCV and did not significantly recover post-SVR, likely due to T cell exhaustion. HIV coinfection did not significantly impact *CD4* expression in coinfected patients post-DAA treatment (**Supplementary Figure S5**). *CD8A* gene expression, a marker of cytotoxic CD8+ T cells, increased with chronic HCV but remain unchanged post-treatment. Individual variability, however, reduced the statistical significance of the findings (**Figure 3D**) (BroChado-Kith et al., 2021; Medrano et al., 2021).

Despite normal transaminases, persistent portal inflammation was seen in 14 out of 17 patients post-DAA (82.3%, 95%CI: 64.2% - 100%) (**Table 1**). Some studies have reported that about 10% of the patients with ongoing inflammation will still have mildly abnormal liver enzymes after treatment (Welsch et al., 2017).

In parallel, spectral imaging of a separate subset revealed heterogeneity in hepatic T cell populations. Inter-patient variability was a major factor contributing to the lack of statistical significance when comparing groups, highlighting the importance of considering patient-level differences in this analysis and the partial overlap with the gene expression cohort. Prior work linked CD3+CD45RO+ memory T cells with fibrosis progression in metabolic dysfunction-associated steatotic liver disease/metabolic dysfunction-associated steatohepatitis (MASLD/MASH) (Sanchez et al., 2023), suggesting a role in HCV-related liver disease. Memory T cells (CD45RO+), indicative of antigen-experienced T cells adapting to persistent infection, contributed to this heterogeneity (**Figure 7**). Memory T cells inhibit NLRP3 activation (Beynon et al., 2012), which was upregulated in the pre-hot group (**Supplementary Table S8**).

By analyzing clinical data, histopathology, and gene expression data, we observed a significant positive association between the pre-hot profile and adverse outcomes (OR = 14.0, 95% CI: 1.31-178.5; *p* = 0.049) (**Figures 8A, C**). However, this association was no longer significant after adjusting for baseline fibrosis stage, MHAI, and HIV status (p = 0.328). Baseline MHAI scores (OR = 1.90, 95% CI: 0.72–6.34) and fibrosis stage (OR = 1.65, 95% CI: 0.44–9.97) showed trends toward predicting poor outcomes, but neither reached statistical significance. These findings suggest that greater baseline inflammation and fibrosis may contribute to post-treatment risk, though larger studies are needed to confirm these associations (Mendizabal et al., 2020). The wide confidence intervals for all predictors, including pre-cold and pre-hot profiles (95% CI: 0.18-2123), suggest the lack of statistical significance is not necessarily an absence of biological relationships. Six patients (27.2%) had advanced fibrosis/cirrhosis, two of whom developed end-stage liver disease (ESLD) (pt 14, 20). Three patients (13.6%) died from liver cancer (pt 4 and 19: HCC, pt 10: ChCA), consistent with reports of a 60–70% reduction in liver-related mortality after achieving SVR (Dang et al., 2020) (**Table 1**). Genes upregulated in pre-hot patients (*CXCR4, BCL2, FYN*) (**Figure 4A**) were associated with liver cancer progression, aligning with previous studies (Lin et al., 2014; Hosseini-Khah et al., 2021; Huang et al., 2022), while viral load showed no correlation (data not shown).

A key strength of this study is the use of paired liver biopsies pre- and post-DAA, enabling direct assessment of histopathology, gene and protein changes, and patient-specific immune responses. Analyses accounted for potential confounding comorbidities (fibrosis stage, MHAI, and HIV status). However, the cohort was relatively small, assays were performed on non-overlapping sample subsets, and some tissue was sufficient only for RNA extraction or spectral imaging. Despite these limitations, the dataset provides a unique and comprehensive view of hepatic immune dynamics, capturing meaningful biological variability among study patients. These findings should be viewed as exploratory and hypothesis-generating, underscoring the need for larger studies to clarify whether pretreatment molecular signatures can provide prognostic information beyond established histologic markers.

DAA treatment effectively controlled infection, deactivated antiviral pathways, and reduced inflammation in most patients, despite initial variability in gene expression, MHAI scores, and fibrosis stages. This study is among the first to identify heterogeneous macrophage subsets in liver biopsies using multiplex immunofluorescence, aligning with recent single-cell findings. Memory T cells were commonly observed before DAA treatment and in some patients with persistent inflammation following treatment. Despite achieving SVR and normalization of gene expression patterns, patients with elevated baseline inflammation and fibrosis, features that overlap with the molecularly defined pre-hot signature, remain at risk for adverse outcomes following DAA treatment. This underscores the need for advanced risk stratification tools, ideally through future studies that integrate gene and protein expression data with clinical parameters. Until molecular profiling becomes clinically accessible, closer monitoring of such patients can be guided by standard tools. By enabling precise, personalized monitoring, this approach has the potential to revolutionize patient management and significantly improve outcomes for high-risk individuals.

## Data Availability

All data generated or analyzed in this study are included in the article and its **Supplementary Materials**. Additional information is available from the corresponding author(s) upon request.

## Supplementary material

Supplementary Figure 1 CIBERSORT was used to estimate the abundance of cell populations, including macrophages, T cells, B cells, and NK cells, using genes from the PanCancer Immune Panel. The key genes contributing to the prediction and deconvolution of these populations are shown: **(A)** genes critical for cell deconvolution before and after DAA treatment, and **(B)** genes relevant within subclusters (pre-hot, pre-cold, post-hot, post-cold, and controls). Heatmaps were generated using each group’s average Z-score values (range: ± 2), with red indicating high gene expression and blue representing low gene expression.

Supplementary Figure 2 Comparison of clinical data from patients pre- and post-DAA treatment subclustered by gene expression patterns. We compared the clinical and laboratory data in patients pre- and post-DAA therapy with two distinct patterns of gene expression and clustering: Pre-hot (high gene expression profile pre-DAA therapy compared to controls), pre-cold (low gene expression profile pre-DAA therapy compared to controls), post-hot (high gene expression post-DAA therapy comparable to pre-hot), and post-cold (low gene expression post-DAA therapy comparable to controls). The pre-hot subcluster showed an increased MHAI score, ALT, and AST, which decreased post-DAA treatment. Liver biopsies were graded for inflammatory activity using the MHAI scoring criteria (0-18) and staged for fibrosis using the Ishak (1-6) criteria (Ishak et al., 1995). Histological and laboratory data were analyzed using limma (R/Bioconductor), accounting for paired and unpaired samples with duplicateCorrelation function; linear models with empirical Bayes moderation were used, and p-values were adjusted by the B-H FDR method. pre, (pre-DAA treatment); post, (post-DAA treatment); MHAI, modified hepatitis activity index; ALT, Alanine transaminase; AST, Aspartate aminotransferase; ALP, Alkaline phosphatase; FFPE, Formalin-fixed paraffin-embedded; PT, Prothrombin time; INR, International Normalized Ratio.

Supplementary Figure 3 **(A-F)** Volcano plots illustrate differentially expressed genes in patients before and after DAA treatment. Volcano plots show log2 fold change versus –log10 adjusted p-value (Benjamini–Yekutieli). Horizontal dashed lines indicating –log10 B-Y adjusted p < 0.05 and vertical dashed lines representing log2 fold change < –0.6 or > 0.6. Red dots denote statistically significantly differentially expressed mRNAs. Abbreviations: Ctrls (controls), pre-hot (high gene expression profile pre-DAA therapy), pre-cold (low gene expression profile pre-DAA therapy), post-hot (high gene expression post-DAA therapy), and post-cold (low gene expression post-DAA therapy).

Supplementary Figure 4 **(A-E)** The Venn diagram illustrates genes significantly expressed across pre- and post-DAA therapy subcluster groups (pre-hot, pre-cold, and post-hot) compared to the control group. The post-cold subcluster group was excluded due to the absence of significantly expressed genes relative to the control. Genes were selected based on an adjusted Benjamini-Yekutieli (B-Y) p-value < 0.05 and a log2-fold change < –0.6 or > 0.6. Genes in red represent upregulated expression, while those in blue indicate downregulated expression. Pre-hot, high gene expression profile pre-DAA therapy; pre-cold, low gene expression profile pre-DAA therapy; post-hot, high gene expression post-DAA therapy.

Supplementary Figure 5 Gene expression patterns in patients with HCV were not significantly affected by their HIV status. **(A)** Box plots comparing the gene expression of *CD4, CD8A*, and *FoxP3* before and after DAA treatment revealed no significant differences between HIV-coinfected and non-coinfected patients. **(B, C)** To determine whether HIV coinfection was a confounding factor in the observed gene expression differences before and after treatment, we conducted a PCA **(B)** and a Fisher’s exact test analysis **(C)** on groups of patients with HCV pre- (pre-hot, n = 9; pre-cold, n = 8) and post- (post-hot, n = 3; post-cold, n = 11) treatment. PCA demonstrated overlapping clustering between HIV-coinfected and non-coinfected patients, both pre- and post-treatment, with clustering influenced primarily by “hot” or “cold” status, as previously described. Fisher’s exact test further confirmed that HIV status did not significantly associate with gene expression patterns pre- or post-treatment (p > 0.05). Normal distribution (Shapiro-Wilk) was assessed, and an unpaired one-way ANOVA or Mann-Whitney test was used to compare the differences between the groups. *p < 0.05; **p < 0.01; ***p < 0.001. PCA of log2-transformed data was used for clustering analysis. The Fisher test assessed gene expression-outcome association (OR >1 = positive association). Ctrl, controls; pre-DAA HIV+, pre-DAA treatment coinfected with HIV; pre-DAA HIV-, pre-DAA treatment without coinfection with HIV; post-DAA HIV+, post-DAA treatment coinfected with HIV; post-DAA HIV-, post-DAA treatment without coinfection with HIV.

## Glossary

AI: AI-artificial intelligence
ALP: alkaline phosphatase
ALT: alanine aminotransferase
ANOVA: Analysis of Variance
AST: aspartate aminotransferase
BMI: body mass index
B-H: Benjamini-Hochberg
B-Y: Benjamini-Yekutieli
CAP: College of American Pathologists
ChCA: cholangiocarcinoma
CLIA: Clinical Laboratory Improvement Amendments
DAA: direct-acting antiviral
DAPI: 4′,6-diamidino-2-phenylindole
DEG: differentially expressed genes
ESLD: end-stage liver disease
FDR: false discovery rate
FFPE: formalin-fixed, paraffin-embedded
H&E: hematoxylin and eosin
HAART: highly active antiretroviral therapy
HCC: hepatocellular carcinoma
HCV: hepatitis C virus
HIV: human immunodeficiency virus
IFI: interferon-inducible protein
IFN: interferon
INR: international normalized ratio
IRB: Institutional Review Board
ISG: interferon-stimulated genes
JAK: Janus kinase
MAIT: mucosal-associated invariant T cells
MASLD: metabolic dysfunction-associated steatotic liver disease
MASH: metabolic dysfunction-associated steatohepatitis
MCL: Markov cluster algorithm
MHAI: modified hepatitis activity index
MHC: major histocompatibility complex
MX: myxovirus resistance
NKC: natural killer cells
NLRP: nucleotide-binding domain, leucine-rich-containing family, pyrin domain containing protein
PCA: principal component analysis
POST: post-DAA treatment
PRE: pre-DAA treatment
Pt: patient
PT: prothrombin time
RCC: resource compiler files
RNA: ribonucleic acid
ROIs: regions of interest
STAT: signal transducer and activation of transcription
STRING: search tool for the retrieval of interacting genes/proteins
SVR: sustained virologic response
TGFB: transforming growth factor beta
TIFF: tagged image file format
TNF: tumor necrosis factor
t-SNE: t-distributed stochastic neighbor embedding
UTMB: University of Texas Medical Branch
