# Supplementary figures and images for: Exploring the liver microenvironment following successful therapy for HCV: gene expression profiling and residual T cell infiltration
Source: Front Cell Infect Microbiol. 2025 Nov 10;15:1662184. doi: 10.3389/fcimb.2025.1662184 (PMC12640901; doi:10.3389/fcimb.2025.1662184)

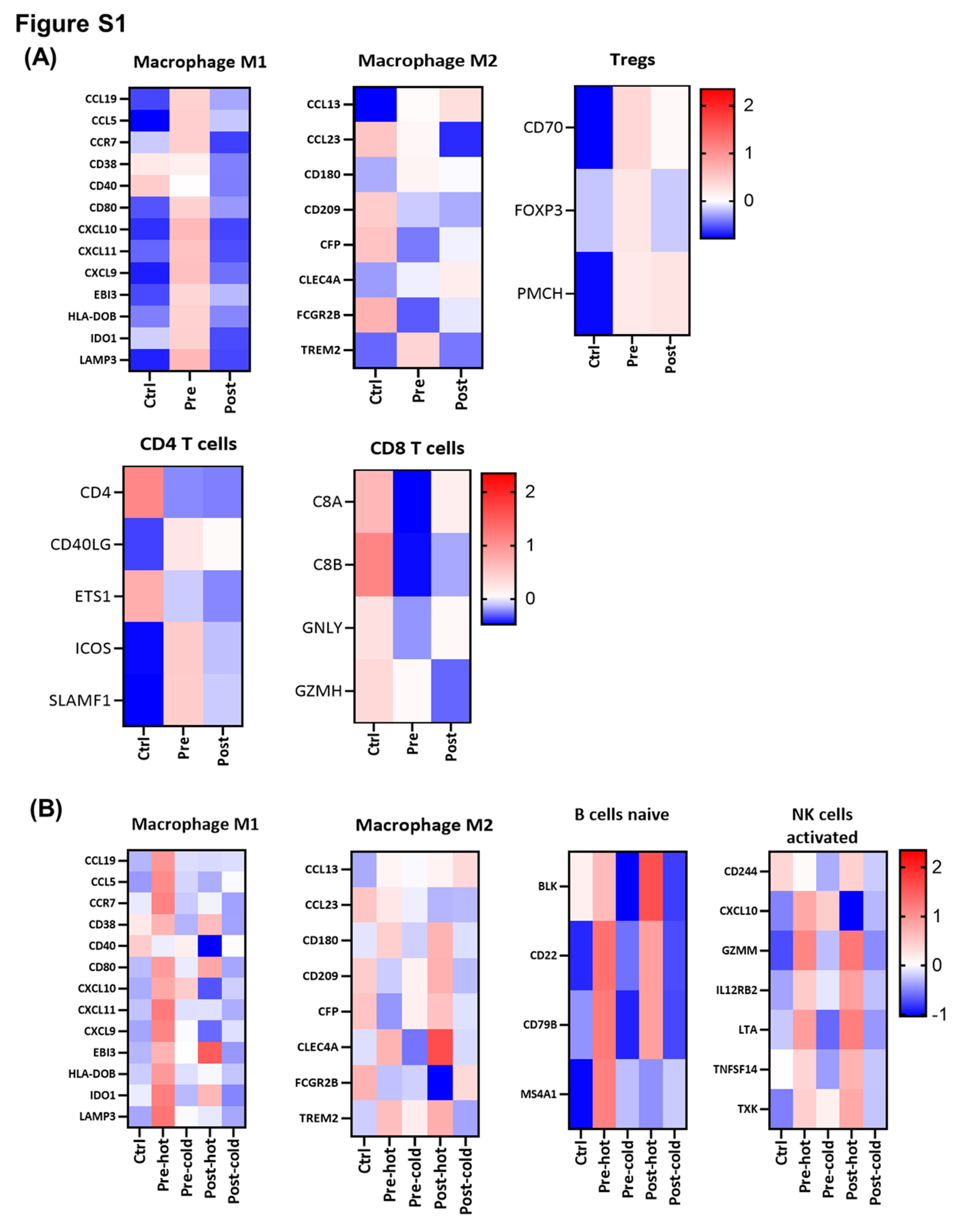

Supplement: Supplementary Figure 1 — CIBERSORT was used to estimate the abundance of cell populations, including macrophages, T cells, B cells, and NK cells, using genes from the PanCancer Immune Panel. The key genes contributing to the prediction and deconvolution of these populations are shown: (A) genes critical for cell deconvolution before and after DAA treatment, and (B) genes relevant within subclusters (pre-hot, pre-cold, post-hot, post-cold, and controls). Heatmaps were generated using each group’s average Z-score values (range: ± 2), with red indicating high gene expression and blue representing low gene expression. [file Image1.tif]

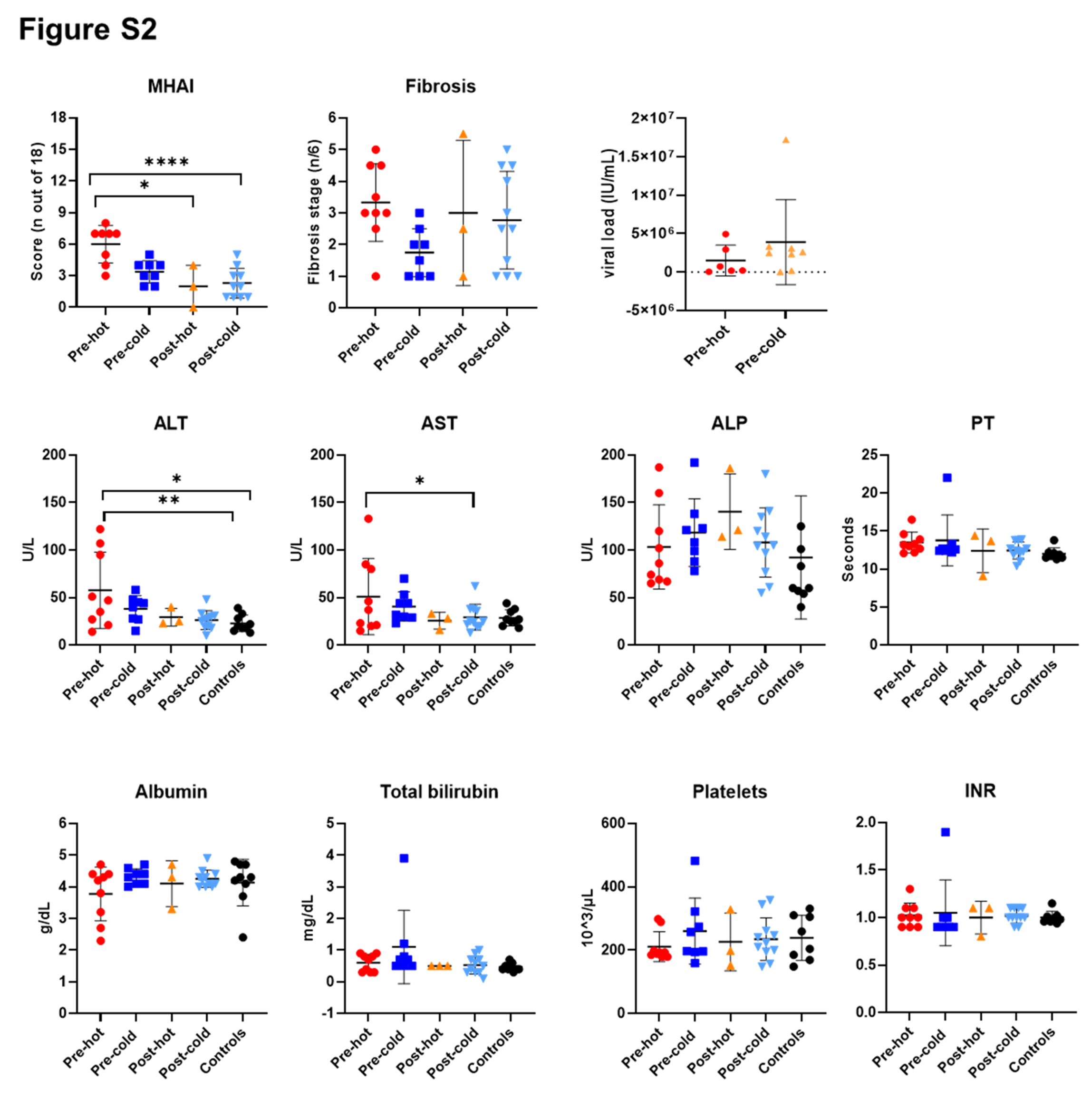

Supplement: Supplementary Figure 2 — Comparison of clinical data from patients pre- and post-DAA treatment subclustered by gene expression patterns. We compared the clinical and laboratory data in patients pre- and post-DAA therapy with two distinct patterns of gene expression and clustering: Pre-hot (high gene expression profile pre-DAA therapy compared to controls), pre-cold (low gene expression profile pre-DAA therapy compared to controls), post-hot (high gene expression post-DAA therapy comparable to pre-hot), and post-cold (low gene expression post-DAA therapy comparable to controls). The pre-hot subcluster showed an increased MHAI score, ALT, and AST, which decreased post-DAA treatment. Liver biopsies were graded for inflammatory activity using the MHAI scoring criteria (0-18) and staged for fibrosis using the Ishak (1-6) criteria (Ishak et al., 1995). Histological and laboratory data were analyzed using limma (R/Bioconductor), accounting for paired and unpaired samples with duplicateCorrelation function; linear models with empirical Bayes moderation were used, and p-values were adjusted by the B-H FDR method. pre, (pre-DAA treatment); post, (post-DAA treatment); MHAI, modified hepatitis activity index; ALT, Alanine transaminase; AST, Aspartate aminotransferase; ALP, Alkaline phosphatase; FFPE, Formalin-fixed paraffin-embedded; PT, Prothrombin time; INR, International Normalized Ratio. [file Image2.tif]

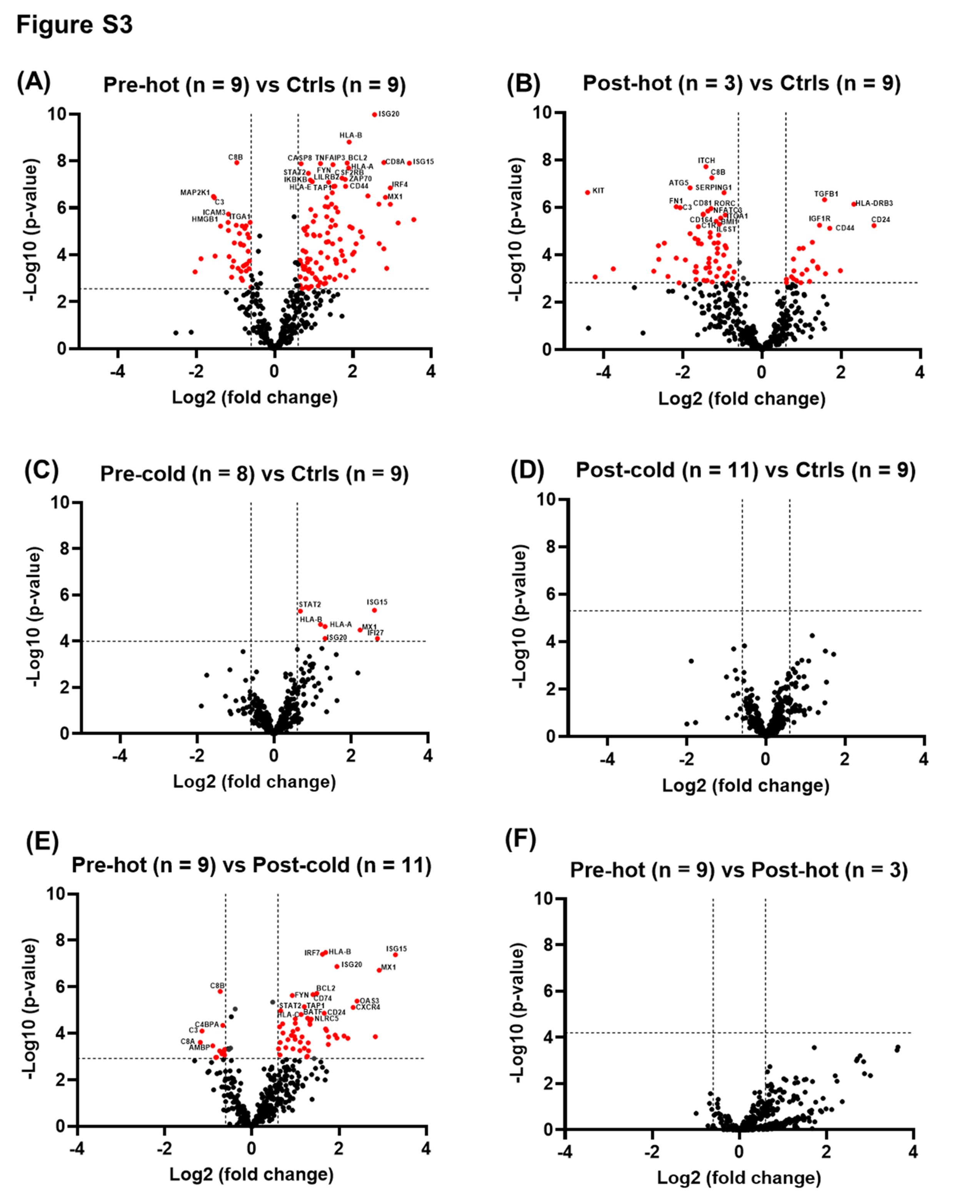

Supplement: Supplementary Figure 3 — (A-F) Volcano plots illustrate differentially expressed genes in patients before and after DAA treatment. Volcano plots show log2 fold change versus –log10 adjusted p-value (Benjamini–Yekutieli). Horizontal dashed lines indicating –log10 B-Y adjusted p < 0.05 and vertical dashed lines representing log2 fold change < –0.6 or > 0.6. Red dots denote statistically significantly differentially expressed mRNAs. Abbreviations: Ctrls (controls), pre-hot (high gene expression profile pre-DAA therapy), pre-cold (low gene expression profile pre-DAA therapy), post-hot (high gene expression post-DAA therapy), and post-cold (low gene expression post-DAA therapy). [file Image3.tif]

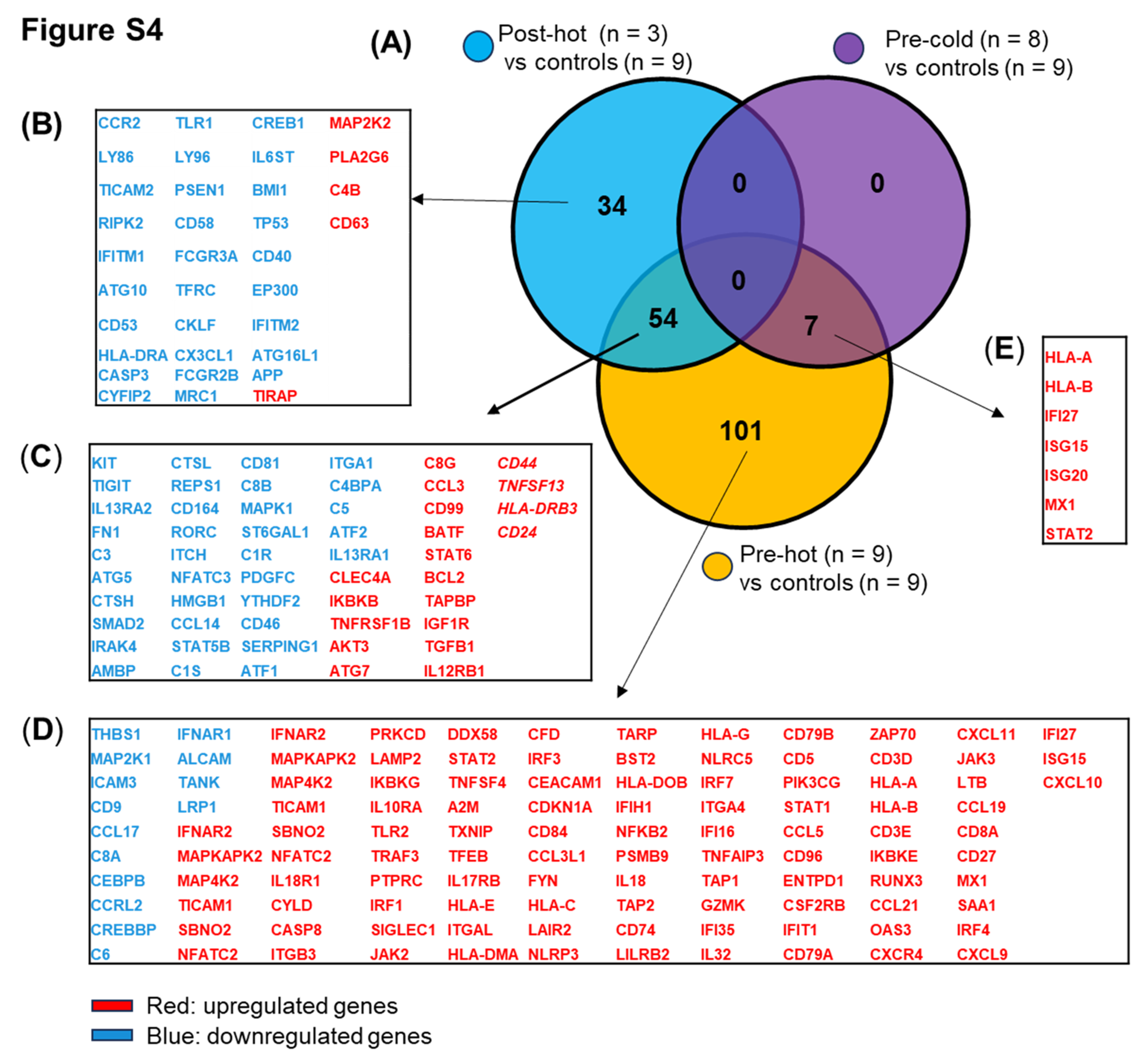

Supplement: Supplementary Figure 4 — (A-E) The Venn diagram illustrates genes significantly expressed across pre- and post-DAA therapy subcluster groups (pre-hot, pre-cold, and post-hot) compared to the control group. The post-cold subcluster group was excluded due to the absence of significantly expressed genes relative to the control. Genes were selected based on an adjusted Benjamini-Yekutieli (B-Y) p-value < 0.05 and a log2-fold change < –0.6 or > 0.6. Genes in red represent upregulated expression, while those in blue indicate downregulated expression. Pre-hot, high gene expression profile pre-DAA therapy; pre-cold, low gene expression profile pre-DAA therapy; post-hot, high gene expression post-DAA therapy. [file Image4.tif]

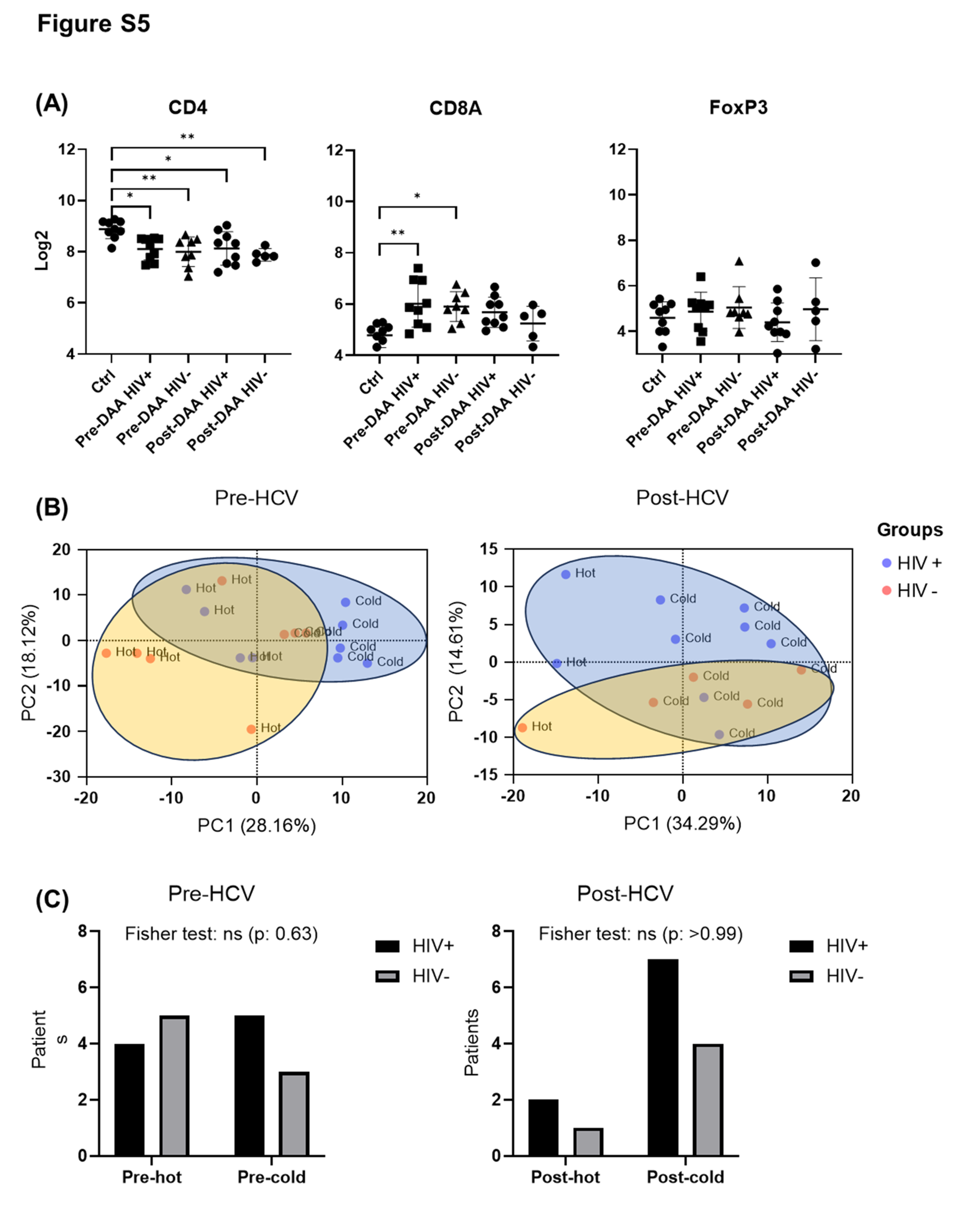

Supplement: Supplementary Figure 5 — Gene expression patterns in patients with HCV were not significantly affected by their HIV status. (A) Box plots comparing the gene expression of CD4, CD8A, and FoxP3 before and after DAA treatment revealed no significant differences between HIV-coinfected and non-coinfected patients. (B, C) To determine whether HIV coinfection was a confounding factor in the observed gene expression differences before and after treatment, we conducted a PCA (B) and a Fisher’s exact test analysis (C) on groups of patients with HCV pre- (pre-hot, n = 9; pre-cold, n = 8) and post- (post-hot, n = 3; post-cold, n = 11) treatment. PCA demonstrated overlapping clustering between HIV-coinfected and non-coinfected patients, both pre- and post-treatment, with clustering influenced primarily by “hot” or “cold” status, as previously described. Fisher’s exact test further confirmed that HIV status did not significantly associate with gene expression patterns pre- or post-treatment (p > 0.05). Normal distribution (Shapiro-Wilk) was assessed, and an unpaired one-way ANOVA or Mann-Whitney test was used to compare the differences between the groups. *p < 0.05; **p < 0.01; ***p < 0.001. PCA of log2-transformed data was used for clustering analysis. The Fisher test assessed gene expression-outcome association (OR >1 = positive association). Ctrl, controls; pre-DAA HIV+, pre-DAA treatment coinfected with HIV; pre-DAA HIV-, pre-DAA treatment without coinfection with HIV; post-DAA HIV+, post-DAA treatment coinfected with HIV; post-DAA HIV-, post-DAA treatment without coinfection with HIV. [file Image5.tif]
