# Supplementary material for: Exploring the liver microenvironment following successful therapy for HCV: gene expression profiling and residual T cell infiltration
Source: Front Cell Infect Microbiol. 2025 Nov 10;15:1662184. doi: 10.3389/fcimb.2025.1662184 (PMC12640901; doi:10.3389/fcimb.2025.1662184)
Supplement: Supplementary file 6 [file Table1.docx]

**Supplementary data**

**Exploring the liver microenvironment following successful therapy for HCV: Gene expression profiling and identification of residual T cell infiltration.**

Daniel E. Millian^1^^┼^, Esteban Arroyave^1┼^, Timothy G. Wanninger^2^, Santhoshi Krishnan^3,4^, Daniel Bao^5^, Jared R. Zhang^5^, Arvind Rao^3,4,6,7,8^, Heidi Spratt^9^, Monique Ferguson^10^, Vincent Chen^11^, Kellen Henning^2^, Heather L. Stevenson^1,5^, and Omar A. Saldarriaga*^1^.

**Table of contents**

**Table S1……………………………………………………………………………………….…...2**

**Table S2……………………………………………………………………………………….…...3**

**Table S3……………………………………………………………………………………….…...4**

**Table S4……………...……………………………………………………………………….…....4**

**Table S5……………………………………………………………………………………...........5**

**Table S6……………………………………………………………………………………...........6**

**Table S7……………………………………………………...…………………………………....7-8**

**Table S8……………………………………...…………………………………………………....9-12**

**Table S9…………...………………………………………………………………………………13-14**

**Table S10……………………………………………………………………...…………………..15**

**Table S11………...……………………………………………………………………………......15**

**Table S12...………………………………………………....…………………………………......16-18**

**Table S13……………………………………………………………………………………….….18**

**Table S1.** Demographic characteristics of controls and comparison with study patients

|  | **Controls n=15**  **(n, (%))** | **Pre-DAA n=22**  **(n, (%))** | **Post-DAA n=17**  **(n, (%))** | **ANOVA p-value** |
| --- | --- | --- | --- | --- |
| ***Age (yrs.), mean*** ± *S.D.* | 53.8 ± 12.9 | 52.45 ± 6.58 | 54.82 ± 7.82 | NS |
| *Sex, n (%)* |  |  |  |  |
| Men | 8 (54) | 14 (64) | NA |  |
| Women | 7 (46) | 8 (36) | NA | NS |
| ***Race*** |  |  |  |  |
| White | 11 (80) | 16 (64.29) | NA |  |
| Non-White | 4 (20) | 6 (35.71) | NA | NS |
| ***Ethnicity*** |  |  |  |  |
| Hispanic | 3 (20) | 5 (23) | NA |  |
| Non-Hispanic | 12 (80) | 17 (77) | NA | NS |
| **BMI** | 26.99 ± 7.46 | 28.22 ± 6.09 | 29.70 ± 6.97 | NS |

**Abbreviations:** Pre-DAA, pre-treatment; post-DAA, post-treatment. NA, not applicable.

NS, not significant; *p < 0.05; **p < 0.01; ***p < 0.001; ****p < 0.0001.

**Table S2.** geNorm-identified stable housekeeping genes for normalization (PanCancer Immune Panel).

| **Gene Name** | **Order selected by geNorm** | **SD after normalization** |
| --- | --- | --- |
| **MRPS5** | 1 | 0.241 |
| **TBP** | 2 | 0.26 |
| **COG7** | 3 | 0.172 |
| **HDAC3** | 4 | 0.237 |
| **SAP130** | 5 | 0.281 |
| **AGK** | 6 | 0.341 |
| **PRPF38A** | 7 | 0.3 |
| **EIF2B4** | 8 | 0.313 |
| **CNOT10** | 9 | 0.273 |
| **TMUB2** | 10 | 0.218 |
| **ABCF1** | 11 | 0.296 |
| **MTMR14** | 12 | 0.22 |
| **CNOT4** | 13 | 0.25 |
| **FCF1** | 14 | 0.255 |
| **SF3A3** | 15 | 0.236 |
| **ZNF143** | 16 | 0.293 |
| **EDC3** | 17 | 0.288 |
| **SDHA** | 18 | 0.33 |
| **DDX50** | 19 | 0.295 |
| **DHX16** | 20 | 0.33 |
| **ZKSCAN5** | 21 | 0.331 |
| **TLK2** | 22 | 0.324 |
| **DNAJC14** | 23 | 0.339 |
| **TRIM39** | 24 | 0.351 |
| **GUSB** | 25 | 0.365 |
| **AMMECR1L** | 26 | 0.369 |
| **PPIA** | discarded | 0.371 |
| **ZC3H14** | discarded | 0.376 |
| **POLR2A** | discarded | 0.454 |
| **G6PD** | discarded | 0.468 |
| **ALAS1** | discarded | 0.658 |
| **ZNF346** | discarded | 0.425 |
| **ERCC3** | discarded | 0.494 |
| **CC2D1B** | discarded | 0.582 |
| **TUBB** | discarded | 0.586 |
| **NUBP1** | discarded | 0.442 |
| **USP39** | discarded | 0.435 |
| **GPATCH3** | discarded | 0.627 |
| **NOL7** | discarded | 0.598 |
| **HPRT1** | discarded | 0.534 |

**Table S3.** Antibodies and optimized multiplex conditions used to identify human macrophage phenotypes and druggable targets in formalin-fixed, paraffin-embedded liver biopsy tissues.

| **Antibody** | **Vendor/Clone/Isotype** | **Dilution** | **Incubation Time (min)** | **Antigen Retrieval Buffer** | **Opal/**  **Dilution** | **Reaction Position** |
| --- | --- | --- | --- | --- | --- | --- |
| **CD68** | Biogenex/KP1/mouse-IgG1κ | RTU* | 60 | AR9 (Akoya) | 520/1:300 | 1 |
| **MAC387** | Dako/Mac387/mouse-IgG1κ | 1:200 | 30 | AR6 (Biogenex) | 690/1:100 | 2 |
| **CD16** | Abcam/EPR16784/rabbit-IgG | 1:500 | 60 | AR6 (Biogenex) | 620/1:600 | 3 |
| **CD14** | Abcam/EPR3653/rabbit-IgG | 1:500 | 45 | AR6 (Biogenex) | 540/1:600 | 4 |
| **CD163** | Leica/10D6/mouse-IgG1 | RTU* | 30 | AR6 (Biogenex) | 650/1:100 | 5 |
| **DAPI** | Akoya | 15µL/mL | 4 | AR6 (Biogenex) | - | 6 |

**Table S4.** Antibodies and optimized multiplex conditions used to identify human T cell phenotypes in formalin-fixed, paraffin-embedded liver biopsy tissues.

| **Antibody** | **Vendor/Clone/isotype** | **Dilution** | **Incubation Time (min)** | **Antigen Retrieval**  **Buffer** | **Opal/**  **Dilution** | **Reaction Position** |
| --- | --- | --- | --- | --- | --- | --- |
| **CD8** | Leica/4B11/IgG2b | 1:4 | 45 | AR9 (Akoya) | 570/1:200 | 1 |
| **CD4** | Leica/4B12/IgG1 | RTU | 60 | AR9 (Akoya) | 520/1:50 | 2 |
| **CD3** | Leica/LN10/IgG1 | RTU | 45 | AR6 (Biogenex) | 650/1:100 | 3 |
| **FoxP3** | CST/D608R/IgG | 1:250 | 30 | AR6 (Biogenex) | 620/1:250 | 4 |
| **CD45RO** | Leica/UCHL1/IgG2a | RTU | 45 | AR6 (Biogenex) | 690/1:100 | 5 |
| **DAPI** | Akoya | 15µL/mL | 5 | AR6 (Biogenex) | - | 6 |

**Table S5-S13: nCounter (NanoString) gene expression results.** Gene expression analysis was performed in patients with HCV pre-DAA treatment (n=17), post-DAA treatment (n=14) and controls (n = 9) using the PanCancer immune profiling panel, the nCounter sprint profiler and the nSolver advanced analysis module. Log2 fold change and the adjusted (p < 0.05) are shown.

**Table S5.** HCV patients pre-DAA treatment (n=17) compared to controls (n=9) (volcano plot Figure 2C)

|  | **Log2 fold change** | **Lower confidence limit (log2)** | **Upper confidence limit (log2)** | **P-value** | **BY.p.value (p<0.05)** |
| --- | --- | --- | --- | --- | --- |
| **ISG15** | 3.11 | 2.28 | 3.93 | 9.34E-09 | 0.0000236 |
| **HLA-B** | 1.61 | 1.17 | 2.05 | 1.68E-08 | 0.0000236 |
| **STAT2** | 0.778 | 0.557 | 1 | 4.00E-08 | 0.0000328 |
| **HLA-A** | 1.65 | 1.17 | 2.12 | 4.68E-08 | 0.0000328 |
| **MX1** | 2.58 | 1.79 | 3.37 | 1.87E-07 | 0.000105 |
| **HLA-E** | 0.805 | 0.538 | 1.07 | 8.33E-07 | 0.000389 |
| **IFI27** | 2.95 | 1.95 | 3.96 | 1.36E-06 | 0.000545 |
| **IFI35** | 1.41 | 0.908 | 1.92 | 3.14E-06 | 0.0011 |
| **BST2** | 1.08 | 0.692 | 1.47 | 3.81E-06 | 0.00119 |
| **CASP8** | 0.513 | 0.32 | 0.705 | 7.21E-06 | 0.00202 |
| **HLA-DRB3** | 1.49 | 0.892 | 2.08 | 1.93E-05 | 0.00458 |
| **IFI16** | 1.13 | 0.677 | 1.59 | 2.08E-05 | 0.00458 |
| **IFNAR2** | 0.407 | 0.242 | 0.573 | 2.37E-05 | 0.00458 |
| **PSMB9** | 1.16 | 0.69 | 1.64 | 2.48E-05 | 0.00458 |
| **CD44** | 1.21 | 0.714 | 1.7 | 2.56E-05 | 0.00458 |
| **IFIT1** | 1.68 | 0.992 | 2.36 | 2.61E-05 | 0.00458 |
| **CXCL10** | 3.06 | 1.8 | 4.31 | 2.84E-05 | 0.00468 |
| **LAMP2** | 0.608 | 0.352 | 0.864 | 3.99E-05 | 0.00622 |
| **CXCL9** | 2.33 | 1.33 | 3.33 | 5.39E-05 | 0.00796 |
| **ITGAL** | 0.783 | 0.442 | 1.12 | 6.38E-05 | 0.00894 |
| **HLA-C** | 1.09 | 0.616 | 1.57 | 6.88E-05 | 0.00918 |
| **OAS3** | 1.79 | 0.972 | 2.6 | 0.000121 | 0.0148 |
| **C8G** | 0.776 | 0.422 | 1.13 | 0.000121 | 0.0148 |
| **STAT5B** | -0.727 | -1.07 | -0.385 | 0.000177 | 0.0204 |
| **TNFRSF1B** | 0.688 | 0.363 | 1.01 | 0.000187 | 0.0204 |
| **CD24** | 1.84 | 0.968 | 2.71 | 0.00019 | 0.0204 |
| **MAP2K1** | -1.04 | -1.53 | -0.545 | 0.000203 | 0.0204 |
| **ICAM3** | -0.83 | -1.22 | -0.435 | 0.000204 | 0.0204 |
| **CYLD** | 0.594 | 0.309 | 0.879 | 0.000223 | 0.0216 |
| **THBS1** | -1.89 | -2.81 | -0.976 | 0.000252 | 0.0234 |
| **IL32** | 1.34 | 0.689 | 1.99 | 0.000259 | 0.0234 |
| **TAPBP** | 0.947 | 0.479 | 1.41 | 0.000319 | 0.028 |
| **RPS6** | -0.676 | -1.02 | -0.335 | 0.000409 | 0.0347 |
| **TGFB1** | 0.799 | 0.389 | 1.21 | 0.000493 | 0.0406 |
| **CCL19** | 2.05 | 0.986 | 3.11 | 0.000549 | 0.0439 |

**Table S6.** HCV patients pre-DAA (n = 17) compared to post-DAA (n = 14) treatment (volcano plot Figure 2C)

|  | **Log2 fold change** | **Lower confidence limit (log2)** | **Upper confidence limit (log2)** | **P-value** | **BY.p.value**  **(p<0.05)** |
| --- | --- | --- | --- | --- | --- |
| **ISG15** | 3.01 | 2.29 | 3.73 | 4.85E-09 | 0.0000132 |
| **MX1** | 2.64 | 1.96 | 3.32 | 2.23E-08 | 0.0000304 |
| **HLA-B** | 1.4 | 1 | 1.79 | 1.13E-07 | 0.000102 |
| **OAS3** | 2.34 | 1.64 | 3.04 | 3.85E-07 | 0.000262 |
| **IFIT1** | 1.73 | 1.15 | 2.31 | 2.64E-06 | 0.00144 |
| **IFI27** | 2.09 | 1.29 | 2.9 | 1.93E-05 | 0.00876 |
| **STAT2** | 0.515 | 0.31 | 0.72 | 3.17E-05 | 0.0124 |
| **HLA-A** | 1.12 | 0.664 | 1.57 | 3.93E-05 | 0.0134 |
| **STAT1** | 1.59 | 0.923 | 2.27 | 6.71E-05 | 0.0203 |
| **CXCL10** | 2.66 | 1.5 | 3.83 | 0.00011 | 0.0299 |
| **IFI35** | 1.06 | 0.585 | 1.54 | 0.00015 | 0.0372 |

**Table S7.** HCV patients pre-hot (n = 9) compared to pre-cold (n = 8) (volcano plot Figure 4A)

|  | **Log2 fold change** | **Lower confidence limit (log2)** | **Upper confidence limit (log2)** | **P-value** | **BY.p.value**  **(p<0.05)** |
| --- | --- | --- | --- | --- | --- |
| **CXCR4** | 2.92 | 2.02 | 3.82 | 8.19E-07 | 0.00223 |
| **FYN** | 1.03 | 0.693 | 1.36 | 1.89E-06 | 0.00257 |
| **BCL2** | 1.53 | 1.01 | 2.06 | 4.59E-06 | 0.00373 |
| **CD74** | 1.41 | 0.916 | 1.91 | 6.35E-06 | 0.00373 |
| **C8B** | -0.711 | -0.961 | -0.46 | 6.84E-06 | 0.00373 |
| **SERPING1** | -0.557 | -0.758 | -0.356 | 9.59E-06 | 0.00436 |
| **CD79B** | 1.55 | 0.98 | 2.12 | 1.25E-05 | 0.00488 |
| **LTB** | 2.34 | 1.43 | 3.24 | 2.62E-05 | 0.00893 |
| **CCL3** | 1.07 | 0.653 | 1.49 | 2.95E-05 | 0.00895 |
| **ITGA4** | 1.4 | 0.838 | 1.97 | 4.33E-05 | 0.0118 |
| **IKBKE** | 1.84 | 1.07 | 2.6 | 6.65E-05 | 0.0165 |
| **IRF7** | 1.06 | 0.617 | 1.51 | 7.27E-05 | 0.0165 |
| **CD24** | 1.56 | 0.903 | 2.23 | 8.16E-05 | 0.0171 |
| **IL13RA1** | -0.453 | -0.646 | -0.26 | 8.97E-05 | 0.0175 |
| **NLRC5** | 1.23 | 0.698 | 1.76 | 0.000104 | 0.018 |
| **CSF2RB** | 1.24 | 0.706 | 1.78 | 0.000107 | 0.018 |
| **HLA-DMA** | 0.989 | 0.56 | 1.42 | 0.000112 | 0.018 |
| **JAK3** | 2.21 | 1.24 | 3.18 | 0.000132 | 0.0199 |
| **IL10RA** | 0.932 | 0.505 | 1.36 | 0.000213 | 0.0259 |
| **ENTPD1** | 2.14 | 1.15 | 3.13 | 0.000228 | 0.0259 |
| **C4BPA** | -0.629 | -0.919 | -0.339 | 0.00023 | 0.0259 |
| **TNFAIP3** | 0.935 | 0.503 | 1.37 | 0.000231 | 0.0259 |
| **CASP8** | 0.383 | 0.206 | 0.559 | 0.000231 | 0.0259 |
| **C1S** | -0.832 | -1.22 | -0.448 | 0.000234 | 0.0259 |
| **C3** | -1.12 | -1.64 | -0.6 | 0.000242 | 0.0259 |
| **ZAP70** | 1.02 | 0.548 | 1.5 | 0.000247 | 0.0259 |
| **IL2RB** | 1.56 | 0.832 | 2.29 | 0.000263 | 0.026 |
| **ISG20** | 1.23 | 0.657 | 1.81 | 0.000267 | 0.026 |
| **TXNIP** | 0.981 | 0.516 | 1.45 | 0.000311 | 0.0292 |
| **IL7R** | 2.38 | 1.24 | 3.51 | 0.000333 | 0.0303 |
| **BATF** | 0.925 | 0.473 | 1.38 | 0.000427 | 0.0365 |
| **JAK2** | 0.675 | 0.345 | 1 | 0.000428 | 0.0365 |
| **IL12RB1** | 1.33 | 0.678 | 1.99 | 0.000463 | 0.0373 |
| **CD96** | 1.49 | 0.755 | 2.22 | 0.000465 | 0.0373 |
| **C8A** | -1.19 | -1.78 | -0.598 | 0.00052 | 0.0394 |
| **RUNX3** | 1.94 | 0.973 | 2.91 | 0.000531 | 0.0394 |
| **CD8A** | 1.43 | 0.715 | 2.14 | 0.000535 | 0.0394 |
| **IRF4** | 1.87 | 0.933 | 2.8 | 0.000557 | 0.04 |
| **FN1** | -1.11 | -1.68 | -0.55 | 0.000624 | 0.0431 |
| **MAP2K1** | -0.943 | -1.42 | -0.464 | 0.000636 | 0.0431 |
| **SBNO2** | 0.586 | 0.288 | 0.884 | 0.000653 | 0.0431 |
| **ITGA1** | -0.505 | -0.762 | -0.248 | 0.000664 | 0.0431 |
| **LRP1** | -0.288 | -0.436 | -0.141 | 0.000697 | 0.0442 |
| **IKBKB** | 0.546 | 0.265 | 0.828 | 0.000731 | 0.0453 |
| **CXCL9** | 1.94 | 0.936 | 2.94 | 0.000774 | 0.0469 |
| **LAIR2** | 1.08 | 0.519 | 1.64 | 0.000796 | 0.0472 |
| **LILRB2** | 0.829 | 0.397 | 1.26 | 0.000833 | 0.0483 |

**Table S8.** HCV patients pre-hot (n = 9) compared to controls (n = 9) (volcano plot Figures 4C and S4)

|  | **Log2 fold change** | **Lower confidence limit (log2)** | **Upper confidence limit (log2)** | **P-value** | **BY.p.value**  **(p<0.05)** |
| --- | --- | --- | --- | --- | --- |
| **ISG20** | 2.55 | 2 | 3.1 | 1.07E-10 | 0.000000299 |
| **HLA-B** | 1.9 | 1.44 | 2.36 | 1.55E-09 | 0.00000217 |
| **CD8A** | 2.79 | 2.05 | 3.53 | 1.15E-08 | 0.00000450 |
| **C8B** | -0.967 | -1.22 | -0.711 | 1.19E-08 | 0.00000450 |
| **BCL2** | 1.85 | 1.36 | 2.35 | 1.22E-08 | 0.00000450 |
| **ISG15** | 3.44 | 2.53 | 4.36 | 1.25E-08 | 0.00000450 |
| **FYN** | 1.17 | 0.857 | 1.48 | 1.29E-08 | 0.00000450 |
| **CASP8** | 0.677 | 0.497 | 0.858 | 1.30E-08 | 0.00000450 |
| **TNFAIP3** | 1.49 | 1.09 | 1.89 | 1.44E-08 | 0.00000450 |
| **HLA-A** | 1.89 | 1.38 | 2.4 | 2.00E-08 | 0.00000560 |
| **STAT2** | 0.861 | 0.621 | 1.1 | 3.35E-08 | 0.00000854 |
| **CSF2RB** | 1.72 | 1.23 | 2.21 | 5.42E-08 | 0.0000127 |
| **ZAP70** | 1.8 | 1.28 | 2.32 | 6.11E-08 | 0.0000130 |
| **IKBKB** | 0.912 | 0.65 | 1.17 | 6.50E-08 | 0.0000130 |
| **HLA-E** | 0.962 | 0.684 | 1.24 | 7.59E-08 | 0.0000142 |
| **LILRB2** | 1.38 | 0.978 | 1.78 | 8.09E-08 | 0.0000142 |
| **CD44** | 1.54 | 1.08 | 1.99 | 1.17E-07 | 0.0000179 |
| **HLA-DRB3** | 1.81 | 1.27 | 2.34 | 1.21E-07 | 0.0000179 |
| **TAP1** | 1.5 | 1.05 | 1.94 | 1.22E-07 | 0.0000179 |
| **IRF4** | 2.95 | 2.07 | 3.83 | 1.40E-07 | 0.0000197 |
| **IFI16** | 1.47 | 1.02 | 1.92 | 2.27E-07 | 0.0000303 |
| **CD24** | 2.38 | 1.64 | 3.12 | 3.10E-07 | 0.0000394 |
| **MAP2K1** | -1.56 | -2.05 | -1.07 | 3.26E-07 | 0.0000395 |
| **MX1** | 2.83 | 1.95 | 3.72 | 3.57E-07 | 0.0000395 |
| **BATF** | 1.33 | 0.912 | 1.75 | 3.60E-07 | 0.0000395 |
| **C3** | -1.54 | -2.03 | -1.06 | 3.66E-07 | 0.0000395 |
| **LTB** | 2.66 | 1.79 | 3.52 | 6.89E-07 | 0.0000395 |
| **IRF7** | 1.45 | 0.981 | 1.93 | 6.93E-07 | 0.0000678 |
| **CXCL9** | 2.95 | 1.99 | 3.91 | 7.02E-07 | 0.0000678 |
| **BST2** | 1.29 | 0.864 | 1.71 | 8.56E-07 | 0.0000678 |
| **TAP2** | 1.36 | 0.91 | 1.81 | 9.14E-07 | 0.0000800 |
| **CD79B** | 1.56 | 1.04 | 2.07 | 9.77E-07 | 0.0000826 |
| **TNFRSF1B** | 0.926 | 0.616 | 1.23 | 1.16E-06 | 0.0000856 |
| **CCL5** | 1.61 | 1.07 | 2.16 | 1.47E-06 | 0.000121 |
| **ICAM3** | -1.18 | -1.59 | -0.778 | 1.81E-06 | 0.000145 |
| **TGFB1** | 1.04 | 0.679 | 1.4 | 2.14E-06 | 0.000167 |
| **IFNAR2** | 0.498 | 0.325 | 0.671 | 2.36E-06 | 0.000174 |
| **NFKB2** | 1.33 | 0.866 | 1.79 | 2.36E-06 | 0.000174 |
| **CXCL10** | 3.55 | 2.29 | 4.81 | 3.11E-06 | 0.000224 |
| **ITGA4** | 1.46 | 0.941 | 1.98 | 3.34E-06 | 0.000234 |
| **ITGAL** | 0.99 | 0.635 | 1.34 | 3.83E-06 | 0.000262 |
| **ITGA1** | -0.627 | -0.852 | -0.401 | 4.11E-06 | 0.000268 |
| **HMGB1** | -1.19 | -1.62 | -0.762 | 4.12E-06 | 0.000268 |
| **IFI27** | 3.15 | 2.02 | 4.29 | 4.35E-06 | 0.000277 |
| **IFI35** | 1.55 | 0.983 | 2.12 | 5.45E-06 | 0.000335 |
| **ATG5** | -0.981 | -1.34 | -0.622 | 5.50E-06 | 0.000335 |
| **ITCH** | -0.731 | -0.999 | -0.462 | 5.80E-06 | 0.00034 |
| **CD81** | -0.829 | -1.13 | -0.524 | 5.84E-06 | 0.00034 |
| **FN1** | -1.38 | -1.88 | -0.87 | 5.95E-06 | 0.00034 |
| **CD3E** | 1.92 | 1.21 | 2.62 | 6.28E-06 | 0.000348 |
| **NLRC5** | 1.43 | 0.903 | 1.96 | 6.37E-06 | 0.000348 |
| **JAK2** | 0.829 | 0.523 | 1.14 | 6.45E-06 | 0.000348 |
| **IKBKE** | 1.98 | 1.25 | 2.72 | 7.09E-06 | 0.000375 |
| **C4BPA** | -0.762 | -1.05 | -0.478 | 7.49E-06 | 0.000387 |
| **LAMP2** | 0.742 | 0.465 | 1.02 | 7.59E-06 | 0.000387 |
| **REPS1** | -1.18 | -1.63 | -0.735 | 9.02E-06 | 0.00045 |
| **ATG7** | 0.769 | 0.479 | 1.06 | 9.14E-06 | 0.00045 |
| **CXCL11** | 2.19 | 1.36 | 3.03 | 1.03E-05 | 0.000497 |
| **PSMB9** | 1.35 | 0.834 | 1.87 | 1.16E-05 | 0.00055 |
| **STAT5B** | -0.899 | -1.25 | -0.553 | 1.23E-05 | 0.000573 |
| **ATF1** | -0.687 | -0.953 | -0.421 | 1.31E-05 | 0.000604 |
| **SIGLEC1** | 0.826 | 0.505 | 1.15 | 1.37E-05 | 0.000612 |
| **CCL3** | 0.992 | 0.607 | 1.38 | 1.37E-05 | 0.000612 |
| **CD84** | 1.15 | 0.703 | 1.6 | 1.51E-05 | 0.000659 |
| **HLA-DMA** | 1.01 | 0.615 | 1.41 | 1.54E-05 | 0.000661 |
| **LRP1** | -0.38 | -0.529 | -0.232 | 1.56E-05 | 0.000661 |
| **JAK3** | 2.24 | 1.36 | 3.13 | 1.74E-05 | 0.000729 |
| **IFNAR1** | -0.655 | -0.915 | -0.396 | 1.85E-05 | 0.000763 |
| **CD96** | 1.67 | 1 | 2.34 | 2.19E-05 | 0.000888 |
| **CD74** | 1.37 | 0.814 | 1.93 | 2.74E-05 | 0.0011 |
| **IFIH1** | 1.32 | 0.782 | 1.86 | 2.87E-05 | 0.00113 |
| **AMBP** | -1.03 | -1.45 | -0.606 | 3.08E-05 | 0.0012 |
| **CCL19** | 2.67 | 1.57 | 3.77 | 3.28E-05 | 0.00125 |
| **CD164** | -0.874 | -1.23 | -0.514 | 3.32E-05 | 0.00125 |
| **CXCR4** | 2.08 | 1.23 | 2.94 | 3.36E-05 | 0.00125 |
| **IL12RB1** | 1.48 | 0.868 | 2.09 | 3.39E-05 | 0.00125 |
| **RORC** | -0.862 | -1.22 | -0.504 | 3.68E-05 | 0.00134 |
| **CCRL2** | -0.791 | -1.12 | -0.462 | 3.87E-05 | 0.00139 |
| **CD27** | 2.79 | 1.6 | 3.98 | 5.51E-05 | 0.00196 |
| **CD99** | 0.726 | 0.412 | 1.04 | 6.49E-05 | 0.00227 |
| **TNFSF13** | 1.7 | 0.961 | 2.43 | 6.70E-05 | 0.00232 |
| **IL13RA1** | -0.415 | -0.596 | -0.235 | 6.98E-05 | 0.00238 |
| **C1R** | -0.673 | -0.965 | -0.38 | 7.04E-05 | 0.00238 |
| **TRAF3** | 0.791 | 0.446 | 1.14 | 7.52E-05 | 0.00251 |
| **OAS3** | 2.03 | 1.14 | 2.92 | 7.62E-05 | 0.00251 |
| **HLA-C** | 1.18 | 0.665 | 1.7 | 7.98E-05 | 0.0026 |
| **RUNX3** | 1.99 | 1.1 | 2.87 | 9.40E-05 | 0.00303 |
| **IFIT1** | 1.73 | 0.957 | 2.51 | 0.000104 | 0.00331 |
| **KIT** | -1.52 | -2.2 | -0.834 | 0.000112 | 0.00352 |
| **CD9** | -1.16 | -1.68 | -0.633 | 0.000119 | 0.00372 |
| **IL18** | 1.35 | 0.733 | 1.96 | 0.000129 | 0.00398 |
| **IGF1R** | 0.837 | 0.452 | 1.22 | 0.000146 | 0.0044 |
| **IL13RA2** | -1.88 | -2.75 | -1.02 | 0.000146 | 0.0044 |
| **TFEB** | 0.906 | 0.487 | 1.32 | 0.000154 | 0.00458 |
| **IL32** | 1.55 | 0.835 | 2.27 | 0.000156 | 0.00459 |
| **CD5** | 1.56 | 0.836 | 2.29 | 0.000167 | 0.00487 |
| **PIK3CG** | 1.57 | 0.841 | 2.31 | 0.00017 | 0.00487 |
| **SBNO2** | 0.65 | 0.347 | 0.952 | 0.00017 | 0.00487 |
| **CD79A** | 1.79 | 0.955 | 2.63 | 0.000179 | 0.00506 |
| **CD46** | -0.645 | -0.947 | -0.343 | 0.000181 | 0.00508 |
| **CTSH** | -1.05 | -1.55 | -0.559 | 0.000183 | 0.00508 |
| **C8G** | 0.832 | 0.438 | 1.23 | 0.00021 | 0.00574 |
| **MAPKAPK2** | 0.561 | 0.295 | 0.827 | 0.000211 | 0.00574 |
| **CLEC4A** | 0.528 | 0.277 | 0.778 | 0.000214 | 0.00574 |
| **IRF3** | 1.06 | 0.558 | 1.57 | 0.000215 | 0.00574 |
| **CYLD** | 0.668 | 0.349 | 0.986 | 0.00023 | 0.00604 |
| **STAT1** | 1.58 | 0.825 | 2.33 | 0.00023 | 0.00604 |
| **C6** | -0.71 | -1.05 | -0.369 | 0.000246 | 0.00639 |
| **TICAM1** | 0.603 | 0.312 | 0.893 | 0.000255 | 0.00655 |
| **C1S** | -0.756 | -1.12 | -0.39 | 0.000271 | 0.0069 |
| **ST6GAL1** | -0.798 | -1.19 | -0.409 | 0.000296 | 0.00749 |
| **PRKCD** | 0.722 | 0.369 | 1.08 | 0.000305 | 0.00763 |
| **IL10RA** | 0.772 | 0.393 | 1.15 | 0.000315 | 0.0078 |
| **C8A** | -1.08 | -1.61 | -0.548 | 0.000324 | 0.00796 |
| **NLRP3** | 1.19 | 0.599 | 1.78 | 0.000357 | 0.0087 |
| **CTSL** | -0.935 | -1.4 | -0.47 | 0.00037 | 0.00894 |
| **SAA1** | 2.86 | 1.43 | 4.28 | 0.000376 | 0.00901 |
| **PDGFC** | -0.653 | -0.979 | -0.327 | 0.000387 | 0.0092 |
| **LAIR2** | 1.18 | 0.592 | 1.77 | 0.000393 | 0.00926 |
| **STAT6** | 0.738 | 0.368 | 1.11 | 0.000399 | 0.00932 |
| **TXNIP** | 0.882 | 0.44 | 1.32 | 0.000404 | 0.00934 |
| **IKBKG** | 0.771 | 0.384 | 1.16 | 0.000407 | 0.00934 |
| **CCL21** | 2.01 | 0.993 | 3.04 | 0.000462 | 0.0105 |
| **CREBBP** | -0.725 | -1.1 | -0.356 | 0.000489 | 0.011 |
| **TNFSF4** | 0.864 | 0.422 | 1.31 | 0.000506 | 0.0113 |
| **CEBPB** | -0.855 | -1.29 | -0.418 | 0.000506 | 0.0113 |
| **THBS1** | -2.03 | -3.07 | -0.986 | 0.00053 | 0.0117 |
| **TAPBP** | 0.958 | 0.46 | 1.46 | 0.000605 | 0.0132 |
| **ATF2** | -0.363 | -0.553 | -0.174 | 0.00061 | 0.0133 |
| **ENTPD1** | 1.67 | 0.784 | 2.55 | 0.000731 | 0.0158 |
| **NFATC3** | -0.592 | -0.906 | -0.277 | 0.000758 | 0.0162 |
| **NFATC2** | 0.651 | 0.301 | 1 | 0.000867 | 0.0184 |
| **CD3D** | 1.82 | 0.835 | 2.8 | 0.000897 | 0.0187 |
| **HLA-G** | 1.4 | 0.644 | 2.16 | 0.000897 | 0.0187 |
| **CCL17** | -1.1 | -1.69 | -0.505 | 0.000899 | 0.0187 |
| **CFD** | 1.04 | 0.477 | 1.6 | 0.00093 | 0.0192 |
| **TIGIT** | 1.29 | 0.587 | 1.99 | 0.000975 | 0.0199 |
| **CCL14** | -0.854 | -1.32 | -0.388 | 0.001 | 0.0204 |
| **TLR2** | 0.788 | 0.357 | 1.22 | 0.00102 | 0.0206 |
| **PTPRC** | 0.798 | 0.36 | 1.24 | 0.00105 | 0.0211 |
| **GZMK** | 1.53 | 0.686 | 2.37 | 0.0011 | 0.0218 |
| **CEACAM1** | 1.07 | 0.48 | 1.66 | 0.00111 | 0.0218 |
| **AKT3** | 0.53 | 0.237 | 0.823 | 0.00112 | 0.022 |
| **YTHDF2** | -0.563 | -0.875 | -0.251 | 0.00116 | 0.0226 |
| **SMAD2** | -0.821 | -1.28 | -0.363 | 0.00123 | 0.0238 |
| **CCL3L1** | 1.16 | 0.5 | 1.82 | 0.00149 | 0.0286 |
| **HLA-DOB** | 1.3 | 0.554 | 2.04 | 0.0016 | 0.0305 |
| **SERPING1** | -0.363 | -0.572 | -0.154 | 0.00165 | 0.0313 |
| **TARP** | 1.26 | 0.533 | 1.98 | 0.0017 | 0.032 |
| **TANK** | -0.468 | -0.739 | -0.196 | 0.00182 | 0.0341 |
| **IL18R1** | 0.651 | 0.269 | 1.03 | 0.00199 | 0.0369 |
| **MAP4K2** | 0.601 | 0.248 | 0.954 | 0.00204 | 0.0376 |
| **CDKN1A** | 1.11 | 0.457 | 1.77 | 0.00209 | 0.0382 |
| **IRAK4** | 0.563 | 0.231 | 0.896 | 0.00211 | 0.0384 |
| **IL17RB** | 0.933 | 0.38 | 1.49 | 0.0022 | 0.0397 |
| **IRF1** | 0.804 | 0.324 | 1.28 | 0.00232 | 0.0417 |
| **ALCAM** | -0.605 | -0.967 | -0.243 | 0.00237 | 0.0423 |
| **DDX58** | 0.845 | 0.339 | 1.35 | 0.00241 | 0.0427 |
| **MAPK1** | -0.57 | -0.913 | -0.226 | 0.00253 | 0.0446 |
| **C5** | -0.424 | -0.68 | -0.168 | 0.00255 | 0.0446 |
| **A2M** | 0.864 | 0.339 | 1.39 | 0.00272 | 0.0473 |
| **ITGB3** | 0.7 | 0.274 | 1.13 | 0.00274 | 0.0475 |

**Table 9.** HCV patients pre-hot (n = 9) compared to post-cold (n = 11)

|  | **Log2 fold change** | **Lower confidence limit (log2)** | **Upper confidence limit (log2)** | **P-value** | **BY.p.value**  **(p<0.05)** |
| --- | --- | --- | --- | --- | --- |
| **HLA-B** | 1.69 | 1.25 | 2.12 | 3.31E-08 | 0.0000378 |
| **IRF7** | 1.62 | 1.2 | 2.03 | 3.91E-08 | 0.0000378 |
| **ISG15** | 3.29 | 2.44 | 4.15 | 4.16E-08 | 0.0000378 |
| **ISG20** | 1.95 | 1.41 | 2.49 | 1.34E-07 | 0.0000912 |
| **MX1** | 2.92 | 2.09 | 3.74 | 1.92E-07 | 0.000105 |
| **C8B** | -0.724 | -0.956 | -0.492 | 1.56E-06 | 0.000706 |
| **BCL2** | 1.49 | 1.01 | 1.98 | 1.88E-06 | 0.000706 |
| **CD74** | 1.4 | 0.943 | 1.86 | 2.15E-06 | 0.000706 |
| **FYN** | 0.929 | 0.624 | 1.23 | 2.33E-06 | 0.000706 |
| **OAS3** | 2.41 | 1.59 | 3.24 | 4.08E-06 | 0.00111 |
| **CASP8** | 0.477 | 0.313 | 0.641 | 4.55E-06 | 0.00113 |
| **TAP1** | 1.2 | 0.775 | 1.62 | 7.13E-06 | 0.00159 |
| **CXCR4** | 2.32 | 1.5 | 3.15 | 7.57E-06 | 0.00159 |
| **LRP1** | -0.38 | -0.516 | -0.243 | 9.00E-06 | 0.00175 |
| **STAT2** | 0.66 | 0.42 | 0.9 | 1.06E-05 | 0.00193 |
| **CD24** | 1.66 | 1.04 | 2.27 | 1.36E-05 | 0.00231 |
| **BATF** | 1.13 | 0.708 | 1.55 | 1.52E-05 | 0.00244 |
| **IL13RA1** | -0.471 | -0.65 | -0.293 | 1.92E-05 | 0.0029 |
| **NLRC5** | 1.28 | 0.787 | 1.76 | 2.22E-05 | 0.00314 |
| **HLA-C** | 0.996 | 0.613 | 1.38 | 2.39E-05 | 0.00314 |
| **DDX58** | 1.36 | 0.834 | 1.88 | 2.42E-05 | 0.00314 |
| **CD79B** | 1.33 | 0.81 | 1.85 | 2.97E-05 | 0.00368 |
| **IL10RA** | 0.994 | 0.599 | 1.39 | 3.66E-05 | 0.00434 |
| **HLA-E** | 0.705 | 0.424 | 0.987 | 3.85E-05 | 0.00438 |
| **HLA-A** | 1.33 | 0.797 | 1.86 | 4.06E-05 | 0.00443 |
| **C4BPA** | -0.665 | -0.934 | -0.396 | 4.53E-05 | 0.00475 |
| **IKBKB** | 0.638 | 0.377 | 0.898 | 5.18E-05 | 0.00523 |
| **IKBKE** | 1.69 | 0.987 | 2.39 | 6.45E-05 | 0.00617 |
| **ZAP70** | 1.05 | 0.616 | 1.49 | 6.56E-05 | 0.00617 |
| **IFIT1** | 1.71 | 0.991 | 2.43 | 7.46E-05 | 0.00678 |
| **C3** | -1.14 | -1.62 | -0.657 | 7.86E-05 | 0.00692 |
| **TNFAIP3** | 0.943 | 0.544 | 1.34 | 8.12E-05 | 0.00692 |
| **JAK2** | 0.712 | 0.407 | 1.02 | 9.50E-05 | 0.00785 |
| **LTB** | 1.91 | 1.07 | 2.74 | 0.000118 | 0.00946 |
| **HLA-DMA** | 0.906 | 0.51 | 1.3 | 0.000123 | 0.00955 |
| **CXCL9** | 2.11 | 1.19 | 3.04 | 0.000128 | 0.00959 |
| **TXNIP** | 0.976 | 0.546 | 1.41 | 0.000135 | 0.00959 |
| **CXCL10** | 2.83 | 1.58 | 4.09 | 0.000138 | 0.00959 |
| **STAT1** | 1.76 | 0.983 | 2.54 | 0.00014 | 0.00959 |
| **IRF3** | 1.15 | 0.642 | 1.66 | 0.000141 | 0.00959 |
| **CXCL11** | 1.95 | 1.08 | 2.83 | 0.000158 | 0.0105 |
| **IFI27** | 2.2 | 1.22 | 3.18 | 0.000162 | 0.0105 |
| **TAP2** | 0.993 | 0.545 | 1.44 | 0.000179 | 0.0113 |
| **BST2** | 0.828 | 0.453 | 1.2 | 0.000187 | 0.0116 |
| **ITGA4** | 1.11 | 0.599 | 1.63 | 0.000237 | 0.0142 |
| **C8A** | -1.18 | -1.73 | -0.634 | 0.000243 | 0.0142 |
| **SIGLEC1** | 0.647 | 0.347 | 0.947 | 0.000245 | 0.0142 |
| **IL12RB1** | 1.29 | 0.691 | 1.9 | 0.000253 | 0.0144 |
| **SELL** | 1.75 | 0.921 | 2.57 | 3.00E-04 | 0.0167 |
| **AMBP** | -0.896 | -1.32 | -0.468 | 0.000341 | 0.0186 |
| **CCL3** | 0.777 | 0.399 | 1.15 | 0.000409 | 0.0218 |
| **ITGA1** | -0.487 | -0.726 | -0.249 | 0.000432 | 0.0224 |
| **IFI35** | 1.2 | 0.613 | 1.79 | 0.000434 | 0.0224 |
| **IFNAR1** | -0.544 | -0.812 | -0.277 | 0.000461 | 0.0226 |
| **TNFRSF1B** | 0.611 | 0.31 | 0.912 | 0.000463 | 0.0226 |
| **CD84** | 0.944 | 0.479 | 1.41 | 0.000464 | 0.0226 |
| **CD46** | -0.626 | -0.936 | -0.316 | 0.000492 | 0.0233 |
| **TANK** | -0.529 | -0.791 | -0.267 | 0.000495 | 0.0233 |
| **CCRL2** | -0.635 | -0.952 | -0.318 | 0.000546 | 0.0248 |
| **IFIH1** | 1.1 | 0.549 | 1.65 | 0.000546 | 0.0248 |
| **CD8A** | 1.3 | 0.65 | 1.96 | 0.00056 | 0.025 |
| **ALCAM** | -0.737 | -1.11 | -0.367 | 0.000571 | 0.0251 |
| **PDGFC** | -0.647 | -0.977 | -0.317 | 0.00067 | 0.029 |
| **C1S** | -0.69 | -1.05 | -0.334 | 0.000749 | 0.0319 |
| **IRAK4** | 0.644 | 0.308 | 0.981 | 0.000851 | 0.0356 |
| **CD81** | -0.624 | -0.95 | -0.298 | 0.000861 | 0.0356 |
| **CD96** | 1.27 | 0.599 | 1.94 | 0.000951 | 0.0387 |
| **CD3E** | 1.26 | 0.587 | 1.93 | 0.00103 | 0.0414 |
| **HMGB1** | -0.811 | -1.24 | -0.378 | 0.00106 | 0.0419 |
| **MAP2K1** | -0.828 | -1.27 | -0.384 | 0.00108 | 0.0421 |
| **CD5** | 1.43 | 0.658 | 2.21 | 0.0012 | 0.0459 |

**Table S10.** HCV patients pre-cold (n = 8) compared to controls (n = 9) (volcano plot Figures 4C and S4)

|  | **Log2 fold change** | **Lower confidence limit (log2)** | **Upper confidence limit (log2)** | **P-value** | **BY.p.value**  **(p<0.05)** |
| --- | --- | --- | --- | --- | --- |
| **ISG15** | 2.6 | 1.66 | 3.55 | 4.52E-06 | 0.0069 |
| **STAT2** | 0.679 | 0.432 | 0.927 | 4.93E-06 | 0.0069 |
| **HLA-B** | 1.2 | 0.723 | 1.67 | 1.87E-05 | 0.016 |
| **HLA-A** | 1.32 | 0.788 | 1.84 | 2.29E-05 | 0.016 |
| **MX1** | 2.23 | 1.31 | 3.14 | 3.22E-05 | 0.018 |
| **IFI27** | 2.68 | 1.51 | 3.86 | 7.52E-05 | 0.0301 |
| **ISG20** | 1.32 | 0.74 | 1.89 | 7.52E-05 | 0.0301 |

**Table S11.** HCV patients post-hot (n = 3) compared to post-cold (n = 11) (volcano plot Figure 4B)

|  | **Log2 fold change** | **Lower confidence limit (log2)** | **Upper confidence limit (log2)** | **P-value** | **BY.p.value (p<0.05)** |
| --- | --- | --- | --- | --- | --- |
| **SERPING1** | -0.901 | -1.17 | -0.631 | 5.08E-07 | 0.00138 |
| **C8B** | -1.03 | -1.37 | -0.697 | 2.04E-06 | 0.00208 |
| **ITCH** | -1.16 | -1.55 | -0.782 | 2.29E-06 | 0.00208 |
| **KIT** | -4.22 | -5.68 | -2.76 | 5.07E-06 | 0.00345 |
| **NFATC3** | -1.24 | -1.71 | -0.759 | 2.49E-05 | 0.0136 |
| **ATG5** | -1.43 | -2.02 | -0.847 | 5.29E-05 | 0.0166 |
| **CD164** | -1.27 | -1.79 | -0.746 | 5.99E-05 | 0.0166 |
| **C1S** | -1.25 | -1.76 | -0.731 | 6.23E-05 | 0.0166 |
| **CD46** | -1.09 | -1.54 | -0.635 | 6.50E-05 | 0.0166 |
| **C3** | -1.66 | -2.36 | -0.968 | 7.01E-05 | 0.0166 |
| **CD24** | 2.11 | 1.23 | 2.99 | 7.12E-05 | 0.0166 |
| **AMBP** | -1.48 | -2.1 | -0.861 | 7.29E-05 | 0.0166 |
| **BMI1** | -0.913 | -1.3 | -0.527 | 8.17E-05 | 0.0168 |
| **CD40** | -1 | -1.43 | -0.578 | 8.61E-05 | 0.0168 |
| **CD81** | -1.09 | -1.57 | -0.62 | 0.000108 | 0.0197 |
| **ITGA1** | -0.796 | -1.14 | -0.449 | 0.00012 | 0.02 |
| **IRAK4** | -1.55 | -2.24 | -0.871 | 0.00013 | 0.02 |
| **FN1** | -1.72 | -2.48 | -0.963 | 0.000132 | 0.02 |
| **YTHDF2** | -1.05 | -1.52 | -0.585 | 0.000147 | 0.0212 |
| **PDGFC** | -1.11 | -1.61 | -0.616 | 0.000158 | 0.0215 |
| **CFB** | -1.06 | -1.53 | -0.58 | 0.000174 | 0.0226 |
| **MRC1** | -1.13 | -1.65 | -0.618 | 0.000189 | 0.0234 |
| **RORC** | -1.16 | -1.71 | -0.619 | 0.000268 | 0.0318 |
| **CTSH** | -1.58 | -2.33 | -0.825 | 0.000333 | 0.0377 |
| **SMAD2** | -1.43 | -2.12 | -0.747 | 0.000346 | 0.0377 |
| **IL13RA1** | -0.533 | -0.792 | -0.273 | 0.000416 | 0.0436 |
| **TGFB1** | 1.03 | 0.523 | 1.53 | 0.000442 | 0.0446 |

**Table S12.** HCV patients post-hot (n = 3) compared to controls (n = 9) (volcano plot Figures 4C and S4)

|  | **Log2 fold change** | **Lower confidence limit (log2)** | **Upper confidence limit (log2)** | **P-value** | **BY.p.value**  **(p<0.05)** |
| --- | --- | --- | --- | --- | --- |
| **ITCH** | -1.42 | -1.8 | -1.03 | 1.93E-08 | 0.0000540 |
| **C8B** | -1.27 | -1.64 | -0.909 | 5.70E-08 | 0.0000799 |
| **ATG5** | -1.82 | -2.37 | -1.28 | 1.50E-07 | 0.000134 |
| **KIT** | -4.41 | -5.76 | -3.06 | 2.38E-07 | 0.000134 |
| **SERPING1** | -0.962 | -1.26 | -0.667 | 2.40E-07 | 0.000134 |
| **TGFB1** | 1.58 | 1.07 | 2.08 | 4.77E-07 | 0.000223 |
| **HLA-DRB3** | 2.32 | 1.57 | 3.08 | 7.35E-07 | 0.000294 |
| **FN1** | -2.17 | -2.89 | -1.45 | 9.45E-07 | 0.000313 |
| **C3** | -2.07 | -2.75 | -1.38 | 1.03E-06 | 0.000313 |
| **CD81** | -1.29 | -1.73 | -0.862 | 1.12E-06 | 0.000313 |
| **NFATC3** | -1.37 | -1.83 | -0.904 | 1.43E-06 | 0.000365 |
| **RORC** | -1.49 | -2 | -0.978 | 1.92E-06 | 0.000419 |
| **CD164** | -1.49 | -2 | -0.976 | 2.02E-06 | 0.000419 |
| **ITGA1** | -0.93 | -1.25 | -0.609 | 2.09E-06 | 0.000419 |
| **BMI1** | -1.05 | -1.42 | -0.68 | 2.81E-06 | 0.000525 |
| **C1R** | -1.16 | -1.57 | -0.741 | 3.88E-06 | 0.00068 |
| **IL6ST** | -1.08 | -1.47 | -0.684 | 5.07E-06 | 0.000836 |
| **IGF1R** | 1.45 | 0.919 | 1.98 | 5.67E-06 | 0.000864 |
| **CD24** | 2.83 | 1.79 | 3.87 | 5.86E-06 | 0.000864 |
| **AMBP** | -1.61 | -2.2 | -1.01 | 6.47E-06 | 0.000907 |
| **CD44** | 1.71 | 1.07 | 2.35 | 7.60E-06 | 0.00101 |
| **STAT5B** | -1.31 | -1.81 | -0.806 | 1.16E-05 | 0.00148 |
| **CTSH** | -1.82 | -2.52 | -1.12 | 1.28E-05 | 0.00155 |
| **CD46** | -1.1 | -1.53 | -0.671 | 1.48E-05 | 0.00173 |
| **C1S** | -1.31 | -1.83 | -0.794 | 1.79E-05 | 0.00201 |
| **SMAD2** | -1.7 | -2.37 | -1.02 | 2.06E-05 | 0.00222 |
| **IRAK4** | -1.62 | -2.28 | -0.97 | 2.37E-05 | 0.00246 |
| **STAT6** | 1.27 | 0.753 | 1.79 | 2.98E-05 | 0.00289 |
| **YTHDF2** | -1.11 | -1.57 | -0.657 | 3.11E-05 | 0.00289 |
| **IFITM1** | -2.47 | -3.48 | -1.45 | 3.23E-05 | 0.00289 |
| **MAPK1** | -1.21 | -1.7 | -0.71 | 3.24E-05 | 0.00289 |
| **CTSL** | -1.61 | -2.27 | -0.947 | 3.30E-05 | 0.00289 |
| **REPS1** | -1.54 | -2.18 | -0.904 | 3.48E-05 | 0.00296 |
| **ATF1** | -0.936 | -1.33 | -0.545 | 4.13E-05 | 0.00335 |
| **TICAM2** | -2.62 | -3.72 | -1.53 | 4.18E-05 | 0.00335 |
| **EP300** | -0.908 | -1.29 | -0.522 | 5.20E-05 | 0.00393 |
| **CD99** | 1.04 | 0.598 | 1.48 | 5.30E-05 | 0.00393 |
| **PDGFC** | -1.13 | -1.61 | -0.646 | 5.40E-05 | 0.00393 |
| **ATG7** | 0.938 | 0.538 | 1.34 | 5.47E-05 | 0.00393 |
| **MRC1** | -1.13 | -1.63 | -0.63 | 8.64E-05 | 0.00606 |
| **TP53** | -0.97 | -1.4 | -0.536 | 0.000102 | 0.00695 |
| **ATG10** | -2.17 | -3.17 | -1.18 | 0.000139 | 0.00925 |
| **HMGB1** | -1.34 | -1.95 | -0.722 | 0.000142 | 0.00927 |
| **IKBKB** | 0.791 | 0.427 | 1.16 | 0.00015 | 0.00954 |
| **RIPK2** | -2.61 | -3.82 | -1.4 | 0.000157 | 0.00977 |
| **HLA-DRA** | -1.94 | -2.84 | -1.04 | 0.000164 | 0.00999 |
| **CD63** | 1.28 | 0.679 | 1.88 | 0.000185 | 0.0109 |
| **ST6GAL1** | -1.17 | -1.72 | -0.623 | 0.000187 | 0.0109 |
| **ATF2** | -0.576 | -0.85 | -0.303 | 0.000211 | 0.0121 |
| **TFRC** | -1.35 | -1.99 | -0.707 | 0.000222 | 0.0124 |
| **C4BPA** | -0.823 | -1.23 | -0.42 | 0.000306 | 0.0168 |
| **TLR1** | -1.67 | -2.49 | -0.85 | 0.000314 | 0.0169 |
| **BCL2** | 1.4 | 0.713 | 2.09 | 0.000322 | 0.017 |
| **CCL14** | -1.34 | -2 | -0.679 | 0.000338 | 0.0176 |
| **CD58** | -1.42 | -2.12 | -0.714 | 0.000367 | 0.0187 |
| **TAPBP** | 1.41 | 0.709 | 2.12 | 0.000376 | 0.0188 |
| **CREB1** | -1.09 | -1.63 | -0.546 | 0.000382 | 0.0188 |
| **TIGIT** | -3.76 | -5.64 | -1.88 | 0.00039 | 0.0188 |
| **BATF** | 1.13 | 0.563 | 1.7 | 0.000427 | 0.0203 |
| **TNFSF13** | 1.98 | 0.976 | 2.99 | 0.000469 | 0.0219 |
| **AKT3** | 0.803 | 0.395 | 1.21 | 0.000476 | 0.0219 |
| **LY86** | -2.74 | -4.14 | -1.34 | 0.000497 | 0.0224 |
| **CASP3** | -1.7 | -2.57 | -0.832 | 0.000509 | 0.0227 |
| **C5** | -0.706 | -1.07 | -0.343 | 0.00053 | 0.0231 |
| **LY96** | -1.66 | -2.51 | -0.806 | 0.000535 | 0.0231 |
| **CKLF** | -1.32 | -2 | -0.638 | 0.000558 | 0.0237 |
| **IFITM2** | -0.881 | -1.34 | -0.424 | 0.000595 | 0.0249 |
| **IL12RB1** | 1.6 | 0.767 | 2.44 | 0.000633 | 0.0261 |
| **CCL3** | 1.01 | 0.483 | 1.54 | 0.000651 | 0.0264 |
| **CD40** | -0.938 | -1.44 | -0.439 | 0.000776 | 0.0311 |
| **IL13RA2** | -2.38 | -3.65 | -1.1 | 0.00082 | 0.032 |
| **FCGR2B** | -1.15 | -1.77 | -0.536 | 0.000822 | 0.032 |
| **MAP2K2** | 0.744 | 0.345 | 1.14 | 0.000841 | 0.0323 |
| **CCR2** | -4.22 | -6.49 | -1.95 | 0.000855 | 0.0324 |
| **IL13RA1** | -0.47 | -0.726 | -0.214 | 0.000971 | 0.0363 |
| **APP** | -0.749 | -1.16 | -0.341 | 0.000998 | 0.0368 |
| **TNFRSF1B** | 0.793 | 0.359 | 1.23 | 0.00103 | 0.0374 |
| **CLEC4A** | 0.618 | 0.278 | 0.958 | 0.00108 | 0.0386 |
| **CYFIP2** | -1.67 | -2.58 | -0.747 | 0.00111 | 0.0394 |
| **PSEN1** | -1.47 | -2.29 | -0.654 | 0.0012 | 0.0421 |
| **PLA2G6** | 0.869 | 0.385 | 1.35 | 0.00122 | 0.0421 |
| **FCGR3A** | -1.39 | -2.16 | -0.614 | 0.00123 | 0.0421 |
| **C4B** | 1.2 | 0.525 | 1.87 | 0.00134 | 0.0452 |
| **CX3CL1** | -1.26 | -1.97 | -0.548 | 0.0014 | 0.0468 |
| **TIRAP** | 0.615 | 0.266 | 0.964 | 0.00148 | 0.0487 |
| **C8G** | 0.98 | 0.422 | 1.54 | 0.0015 | 0.0487 |
| **ATG16L1** | -0.881 | -1.38 | -0.379 | 0.00152 | 0.0487 |
| **CD53** | -2.1 | -3.29 | -0.902 | 0.00153 | 0.0487 |

**Table S13.** HCV patients post-cold (n 11) compared to controls (n = 9)

|  | **Log2 fold change** | **Lower confidence limit (log2)** | **Upper confidence limit (log2)** | **P-value** | **BY.p.value**  **(p<0.05)** |
| --- | --- | --- | --- | --- | --- |
| **A2M** | 1.17 | 0.67 | 1.67 | 5.59E-05 | 0.157 |
| **ATF1** | -0.543 | -0.794 | -0.293 | 0.000151 | 0.175 |
| **ICAM3** | -0.815 | -1.2 | -0.43 | 0.000203 | 0.175 |
| **CD8A** | 1.5 | 0.779 | 2.22 | 0.00025 | 0.175 |
| **IRF4** | 1.71 | 0.866 | 2.56 | 0.000345 | 0.193 |
| **CSF2RB** | 0.908 | 0.435 | 1.38 | 0.000608 | 0.23 |
| **NLRP3** | 1.08 | 0.514 | 1.64 | 0.000654 | 0.23 |
| **THBS1** | -1.89 | -2.88 | -0.901 | 0.000658 | 0.23 |
| **TNFSF4** | 0.797 | 0.371 | 1.22 | 0.000813 | 0.253 |
| **LILRB2** | 0.684 | 0.297 | 1.07 | 0.00144 | 0.402 |
| **CCL14** | -0.775 | -1.22 | -0.33 | 0.00162 | 0.402 |
| **CD44** | 0.744 | 0.309 | 1.18 | 0.00193 | 0.402 |
| **STAT5B** | -0.559 | -0.886 | -0.231 | 0.00198 | 0.402 |
| **IFI16** | 0.736 | 0.304 | 1.17 | 0.00201 | 0.402 |
| **ITGAL** | 0.572 | 0.233 | 0.912 | 0.00221 | 0.414 |
| **GTF3C1** | 0.57 | 0.227 | 0.914 | 0.00254 | 0.437 |
| **IL18** | 0.963 | 0.372 | 1.55 | 0.00298 | 0.437 |
| **C8G** | 0.612 | 0.236 | 0.988 | 0.003 | 0.437 |
| **CXCL2** | -0.999 | -1.61 | -0.384 | 0.00306 | 0.437 |
| **HLA-DRB3** | 0.828 | 0.317 | 1.34 | 0.00312 | 0.437 |
| **MAP2K1** | -0.733 | -1.19 | -0.272 | 0.00367 | 0.49 |
| **TGFB1** | 0.544 | 0.199 | 0.889 | 0.00389 | 0.495 |
